# Modulation of Biomaterial‐Associated Fibrosis by Means of Combined Physicochemical Material Properties

**DOI:** 10.1002/advs.202407531

**Published:** 2024-12-06

**Authors:** Lisa E. Tromp, Torben A.B. van der Boon, Roderick H.J. de Hilster, Ruud Bank, Patrick van Rijn

**Affiliations:** ^1^ Department of Biomaterials and Biomedical Technology University of Groningen, University Medical Center Groningen FB‐40, A. Deusinglaan 1 Groningen 9713 AV the Netherlands; ^2^ Department of Pathology and Medical Biology University of Groningen, University Medical Center Groningen A. Deusinglaan 1 Groningen 9713 AV the Netherlands

**Keywords:** biomaterial‐associated fibrosis, cell‐material interactions, fibroblast, foreign body response, high‐throughput screening

## Abstract

Biomaterial‐associated fibrosis remains a significant challenge in medical implants. To optimize implant design, understanding the interplay between biomaterials and host cells during the foreign body response (FBR) is crucial. Material properties are known to influence cellular behavior and can be used to manipulate cell responses, but predicting the right combination for the desired outcomes is challenging. This study explores how combined physicochemical material properties impact early myofibroblast differentiation using the Biomaterial Advanced Cell Screening (BiomACS) technology, which assesses hundreds of combinations of surface topography, stiffness, and wettability in a single experiment. Normal human dermal fibroblasts (NHDFs) are screened for cell density, area, and myofibroblast markers α‐smooth muscle actin (α‐SMA) and Collagen type I (COL1) after 24 h and 7 days of culture, with or without transforming growth factor‐beta (TGF‐β). Results demonstrated that material properties influence fibroblast behavior after 7 days with TGF‐β stimulation, with wettability emerging as the predominant factor, followed by stiffness. The study identified regions with increased cell adhesion while minimizing myofibroblast differentiation, offering the potential for implant surface optimization to prevent fibrosis. This research provides a powerful tool for cell‐material studies and represents a critical step toward enhancing implant properties and reducing complications, ultimately improving patient outcomes.

## Graphical abstract

Biomaterial‐associated fibrosis remains a challenge in medical implants. It is shown that combined physicochemical material properties impact the early myofibroblast differentiation using the Biomaterial Advanced Cell Screening (BiomACS) technology with wettability emerging as the predominant factor, followed by stiffness. Combinations are highlighted that show increased cell adhesion while minimizing myofibroblast differentiation, offering the potential for implant surface optimization to prevent fibrosis.

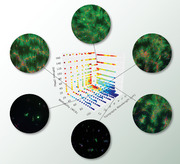

## 1 Introduction

Silicone is widely used in various medical implants and devices, including but not limited to breast implants,^[^ ^1^, ^2^, ^3^ ^]^ cochlear implants,^[^ ^4^ ^]^ intraocular lenses,^[^ ^5^ ^]^ voice prostheses,^[^ ^6^ ^]^ and catheters.^[^ ^7^, ^8^ ^]^ However, biomaterial‐associated fibrosis poses a significant challenge, especially with expanding medical implant use, frequently resulting in complications or implant rejection.^[^ ^10^ ^]^ Fibrosis is the consequence of an excessive foreign body response (FBR), a complex reaction that occurs when a biomaterial is implanted into the body.^[^ ^11^, ^12^, ^13^ ^]^ This response results from the body's natural defense mechanisms recognizing the implanted material as foreign and attempting to eliminate or isolate it. During the FBR, fibroblasts can differentiate into myofibroblasts, characterized by high contractile stress fibers incorporated with high alpha‐smooth muscle actin (α‐SMA) and the production of excessive collagen that forms a fibrous capsule around the implant.^[^ ^14^, ^15^, ^16^ ^]^ This process can ultimately lead to scar tissue formation, contracture, and fibrosis.^[^ ^17^ ^]^ Differentiation of myofibroblasts is a key aspect of fibrosis, as the activated myofibroblasts can create a dysregulation that results in the persistent activation of more myofibroblasts and consequent extracellular matrix (ECM) secretion through a pathological positive feedback loop in which fibroblasts also secrete TGF‐β, further activating more myofibroblast differentiation.^[^ ^14^ ^]^ Hence, by addressing this transformation process, the further development and progression of fibrosis can potentially be inhibited.

To optimize implant design, understanding the interplay between the physicochemical properties of the material and host cells during the FBR is crucial.^[^ ^18^, ^19^, ^20^ ^]^ The field of materiobiology is focused on the manipulation of cell behavior toward desired cellular responses, such as adhesion, proliferation, and differentiation, thereby mitigating complications after medical device implantation.^[^ ^21^, ^22^ ^]^ In this way, the material itself can actively contribute to alleviating biomaterial‐associated fibrosis.^[^ ^23^ ^]^ Within the discovery of biomaterials, researchers have extensively studied implant surface modifications to improve cellular responses to the biomaterial, including alterations in surface topography,^[^ ^24^, ^25^ ^]^ chemistry,^[^ ^26^, ^27^, ^28^ ^]^ and mechanical properties.^[^ ^29^, ^30^, ^31^ ^]^ Surface texturing has been widely used to enhance breast implant stability and the addition of topography has been shown to mediate the FBR of silicone breast implants by altering inflammation and capsule formation.^[^ ^1^, ^32^ ^]^ In addition to surface roughness,^[^ ^33^ ^]^ studies into cell‐biomaterial interactions have explored surface structuring by exploring micropatterns,^[^ ^34^, ^35^ ^]^ grooves,^[^ ^36^, ^37^ ^]^ biomimetic surfaces,^[^ ^38^ ^]^ and predefined combinations of features.^[^ ^39^, ^40^, ^41^ ^]^ Furthermore, the material's chemistry, including modifications such as plasma polymerization, can significantly influence cell behavior by altering protein adsorption.^[^ ^42^ ^]^ This change in protein adsorption can subsequently impact cell signaling pathways and adhesion. Similarly, wettability, which refers to the surface's ability to attract or repel water, can also influence the FBR by affecting protein adsorption and the resulting cell adhesion to the material.^[^ ^43^ ^]^ The stiffness of a biomaterial impacts the forces transmitted to surrounding tissues, and a mismatch in tissue and implant stiffness can lead to mechanical activation of inflammatory cells, such as macrophages, and fibroblasts during the FBR.^[^ ^44^ ^]^ Varying stiffness has been shown to impact cellular adhesion and spreading, which could potentially contribute to the development of fibrosis.^[^ ^45^, ^46^ ^]^ However, in the body, cells are exposed to multiple parameter cues simultaneously. High‐throughput methods offer significant advantages over traditional research methods because they can handle multiple parameters simultaneously and generate large datasets more efficiently.^[^ ^21^, ^47^, ^48^ ^]^ Multiple screening methods have been developed in this field, such as polymer or hydrogel microarrays,^[^ ^49^, ^50^, ^51^ ^]^ topography arrays,^[^ ^39^, ^52^, ^53^ ^]^ and gradient surfaces.^[^ ^54^, ^55^, ^56^ ^]^ We have previously explored various cell‐material interactions by utilizing different gradient surfaces, ranging from modified topography to variations in stiffness and wettability.^[^ ^57^, ^58^, ^59^ ^]^

Here, we explored the influence of combined physicochemical material properties on the early transformation of fibroblasts toward fibrotic myofibroblasts via the Biomaterial Advanced Cell Screening (BiomACS) technology, our recently developed high‐throughput combinatorial cell‐screening technology that combines four double‐orthogonal physicochemical surface parameter gradients.^[^ ^60^ ^]^ The double orthogonal gradients (DOGs) are based on a combination of gradients of surface wrinkled topography (T), stiffness (S), and wettability (W) that are prepared by sequential plasma oxidation treatments of polydimethylsiloxane (PDMS) under a 90‐degree angle to each other. Every position on the surface has a unique combination of the three parameters to which cellular behavior can be assessed, potentially revealing an optimum combination of material properties to prevent the development of biomaterial‐associated fibrosis.

Normal human dermal fibroblasts (NHDFs) were seeded onto the DOG substrates and cultured for 24 h and 7 days in the presence or absence of transforming growth factor beta (TGF‐β) to simulate the inflammatory environment during the FBR.^[^ ^61^ ^]^ After fixation, cells were stained for nucleus, α‐SMA, and Collagen type I (COL1), imaged, and analyzed on each position of the gradients using an automated pipeline. The screening shows a wide range of different phenotypes toward different combinations of the material properties, and the feature importance of the varying parameters was investigated. From all the data points obtained from the screening, “hits” are identified as regions of interest (ROIs) with a predefined combination of material properties that result in a specific cell behavior. Those ROIs or “hits” were verified in translational cell experiments on homogeneous substrates bearing those particular combinations of material properties. The findings of this study show that myofibroblast transformation in the presence of TGF‐β is affected by different physicochemical material combinations, which could aid the development of novel implant surfaces that can actively prevent biomaterial‐associated fibrosis.

## 2 Results

### 2.1 DOG Screening Reveals Diverse Fibroblast Behavior to Surface Material Properties

The DOG screening platform, as reported before,^[^ ^60^ ^]^ consisting of the four DOGs Topography‐Stiffness (T‐S), Topography‐Wettability (T‐W), Topography‐Stiffness|Wettability (T‐S|W), and Stiffness‐Wettability (S‐W), was fully characterized and prepared for cell screening experiments. The gradients of topography, stiffness, and wettability show an increase in wrinkle size, an increase in stiffness, and a decrease in water contact angle from the closed to the open side of the mask, respectively (Figure S1, Supporting Information). NHDFs were seeded onto the DOG samples and cultured for 24 h or 7 days, with or without the addition of TGF‐β, after which the cells were fixated and immunolabelled for α‐SMA and COL1. The entire 20×20 mm^2^ DOG area was captured by imaging with a TissueFAXS automated fluorescent microscope (Figure S2, Supporting Information). Every image within the DOG area was analyzed using a customized CellProfiler pipeline, which segmented individual cells based on nucleus and fluorescent protein expression, followed by quantification of the median cell density, α‐SMA and COL1 expression (corrected total cell fluorescence, CTCF).

**Figure 1:**
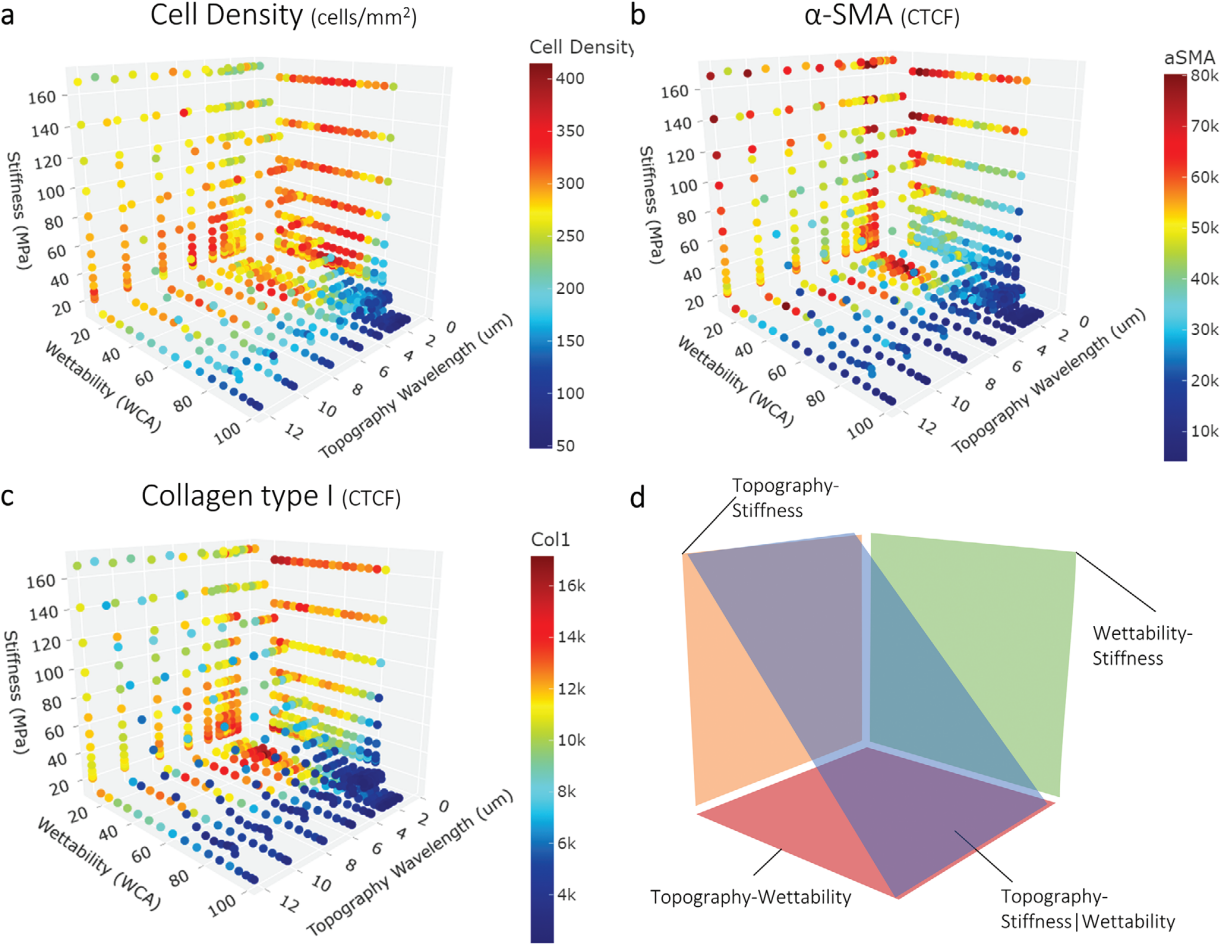
DOG screening overview after 7 days with stimulation of TGF‐β. 3D plots show the mean of the screening replicates (n = 4) as a color scale on the axes of topography, stiffness, and wettability. a) Cell density is quantified as cells mm^−2^. b) α‐SMA expression was quantified as Corrected Total Cell Fluorescence (CTCF, a.u.). c) Collagen type I expression was quantified as CTCF (a.u.). d) Schematic overview of each plane in the 3D box, corresponding to each DOG.

In general, our results show that after 24 h, fibroblasts maintain their native state without expressing α‐SMA and COL1 during their initial adhesion independent of the physicochemical stimulation or the exposure to TGF‐β (Figure S2, Supporting Information). The screening results after 7 days of cell culture do show a large difference in cell density and expression pattern and the quantified data is displayed in 3D heatmaps that summarize all the screening data as different planes in a 3D box of the three parameters topography, stiffness, and wettability (**Figure** **1** for 7 days TGF‐β (+), Figure S3 (Supporting Information) for 7 days TGF‐β (‐)). The four DOGs from which the datapoints originate are schematically shown as planes in Figure 1d. After 7 days without the presence of an inflammatory environment (absence of TGF‐β), only a minimal number of α‐SMA‐positive cells and limited intracellular COL1 expression can be observed, leading to low values for α‐SMA and COL1 CTCF (Figure S3, Supporting Information). This outcome indicates that material properties alone are not enough to trigger a fibrotic response in fibroblasts within the initial 7 days. However, after 7 days in the presence of TGF‐β, depending on the material surface properties, cells can transform into myofibroblasts that express α‐SMA and COL1. A wide distribution of phenotypes was observed, indicating regions with high cell density and myofibroblast transformation and regions with low cell density and few transformed cells (Figure 1). This difference indicates that in the presence of an inflammatory environment, material properties can be used to delegate the fibroblast response during the FBR. It can be noted that, overall, data points with a WCA > 80° have a low cell density, as well as low expression of α‐SMA and COL1. Contrarily, data points with a WCA < 30° have a high cell density and elevated α‐SMA and COL1 expression. As our results indicate that fibroblasts only transform into myofibroblasts in the presence of the inflammatory environment, the following sections will describe the results found from the timepoint at 7 days and with stimulation of TGF‐β and with α‐SMA as the main marker, although the expression of COL1 follows a similar trend. Results of the other timepoints and conditions (7 days TGF‐β (‐) and 24 h TGF‐β (‐) and (+)) can be found in the Supplementary Information when indicated.

Summarized, these findings show that the DOG screening platform revealed diverse fibroblast behaviors in response to the different material properties, especially after 7 days with TGF‐β stimulation, where cells displayed varying degrees of myofibroblast differentiation.

### 2.2 Material Properties Influence α‐SMA Expression of Fibroblasts When Stimulated with TGF‐β

**Figure 2:**
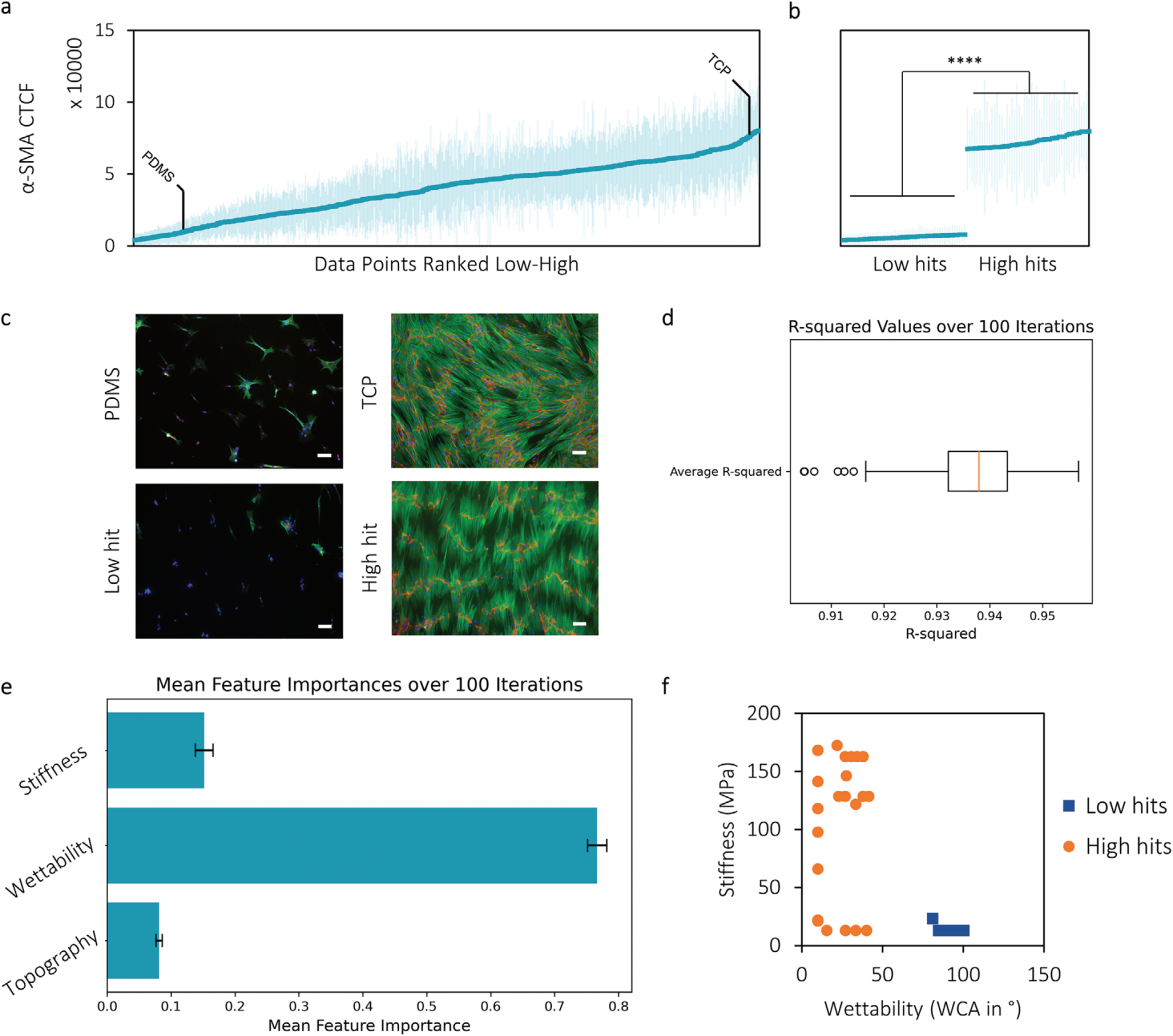
a) Screening results of fibroblasts after 7 days stimulated with TGF‐β ranked from low to high α‐SMA expression (average of n = 4). b) The 50 lowest and 50 highest hits were compared and shown statistically significant (p<0.0001). c) Representative fluorescence images of cells on the low hit and high hit surfaces, stained for nucleus (blue), α‐SMA (green), and COL1 (red). Scale bar is 100 µm. d and e) A machine learning model to predict α‐SMA CTCF was trained with 10‐fold cross‐validation using a random forest algorithm. d) The average R‐squared value with standard deviation of 100 predictive models based on the surface features. e) The mean feature importances with standard deviation over the 100 different iterations was determined to determine the importance for predicting α‐SMA expression in cells. f) surfaces that are indicated as both low and high hits can be distinguished by their wettability and stiffness.

The expression profile of α‐SMA and COL1 follow a similar trend and as α‐SMA is an initial main marker, for clarity, we chose to focus on α‐SMA in the following section but the data of COL1 is presented in the Supporting Information when indicated. By sorting all quantifications of the median α‐SMA CTCF on each location of the DOGs from low to high, we can determine that the data is distributed between 5000 and 80 000 CTCF (A.U.), showing a more than 15‐fold increase in α‐SMA expression between the 50 lowest and highest hits (**Figure** **2**a,b). This difference indicates that by altering the material surface properties topography, stiffness, and wettability, it is possible to obtain very different expression profiles of α‐SMA from the cells. What can be seen from the fluorescence images (Figure 2c), corresponding to a high and low “hit”, is that cells on high “hit” surfaces and on TCP show elongated stress fibers of α‐SMA as well as collagen deposition. Contrarily, untreated PDMS and low “hit” surfaces show less adhered cells and only few α‐SMA positive cells with low COL1 expression.

A machine learning model was trained with 10‐fold cross‐validation that employs a random forest (RF) regression algorithm. This model is able to detect non‐linear relationships in the dataset, enabling to determine correlations between the individual material properties and α‐SMA expression. The R‐squared of the obtained model was assessed on a held‐out test data set consisting of 25% of the data that was not used for training. Model training and testing was performed 100 times with random splitting of the data to calculate the average R‐squared value (0.94 ± 0.01, Figure 2d), which indicates that 94% of the variability in the α‐SMA CTCF can be explained by the model, with a mean absolute error (MAE) of 3462 ± 211 CTCF (< 5% of the total distribution). Additionally, the feature importance of the material properties was quantified (Figure 2e), which gives insight into the contribution of each feature toward the predicted α‐SMA expression. Wettability demonstrated the highest importance on the prediction of the model, followed by a mild effect of stiffness. By showing the 50 lowest and 50 highest values according to their wettability and stiffness (Figure 2f), these findings can be additionally visualized, as it shows that all low hits are found on surfaces with a WCA > 60° and low stiffness. The high hits can be found on surfaces with WCA < 50°, with more than half found on the stiffer surfaces.

Thus, material properties significantly influence α‐SMA expression in fibroblasts when stimulated with TGF‐β, of which the expression varies widely and can lead to extremes in behavior. A random forest regression model identified wettability as the most influential factor, followed by stiffness.

### 2.3 Material Properties Influence Cell Adhesion and Early Fibrosis Markers

**Figure 3:**
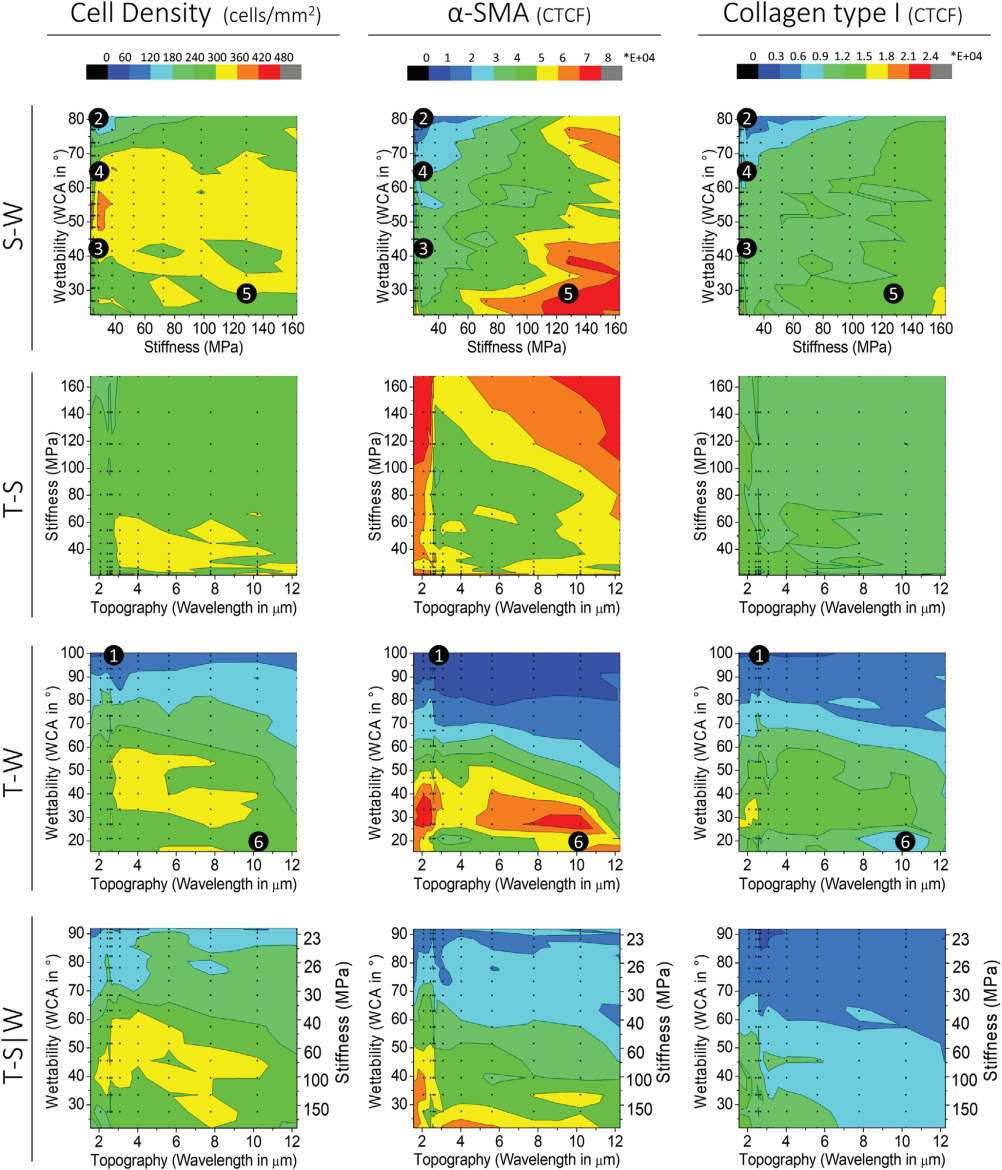
Heatmaps showing the cell screening results after 7 days with stimulation of TGF‐β on each DOG. The average value (n = 4) of cell density in cells mm^−2^, α‐SMA CTCF, and COL1 CTCF was shown on each of the DOGs, each small dot representing a measured data point. Six regions of interest (ROIs) are indicated with the numbered black dots, which were later used for translation experiments on homogeneous samples.

To further explore the observed trends per DOG instead of the dataset as a whole, 2D versions of the heatmaps were prepared that illustrate each quantified value on their respective position on each of the DOGs separately (**Figure** **3** for 7 days TGF‐β (+), Figures S4–S6 for 7 days TGF‐β (‐) and 24 h TGF‐β (+) and (‐)). In the following section, only the results after 7 days with supplementation of TGF‐β will be discussed since this condition shows the most significant differences in cell behavior and relevance to fibrosis development, but the other timepoints and conditions are included in the Supporting Information to still provide the entire dataset. The heatmaps shown in Figure 3 provide an overview of the four separate DOGs, each consisting of a topography, stiffness, or wettability gradient on each axis, with the color scale indicating the specific biological behavior of the cells on each specific location of the gradients (cell density, α‐SMA, and COL1). These figures allow for easy assessment of the difference in cell behavior within the same DOG. For example, it is clear that on each DOG consisting of a wettability gradient (S‐W, T‐W, and T‐S|W), there is an increased cell density, α‐SMA, and COL1 expression on the more hydrophilic side (WCA < 70°) of the DOGs. It can also visually be observed that besides wettability, an increased stiffness is associated with an increased α‐SMA expression (Figure 3, middle column) on the S‐W, T‐S and T‐S|W DOG, and an increased COL1 expression (Figure 3, right column) on the S‐W and T‐S|W DOG. However, this effect of stiffness is not observed for cell density (Figure 3, left column), which mainly seems affected by wettability, ranging from ≈50 cells mm^−2^ on the more hydrophobic side of the DOGs to more than 300 cells mm^−2^ on the hydrophilic side of the DOGs. Moreover, it can be observed that when looking at specific ranges of wettability and stiffness, varying topography can be used to further finetune the biological response.

For example, on the T‐W DOG, it appears that wettability is the most dominant factor while altering topography does not alter α‐SMA expression as much, specifically on the more hydrophobic side of WCA > 80°. However, within the specific WCA of 30°, a topography of ≈4 µm shows lower α‐SMA expression compared to a topography of 10 µm, indicating that topography contributes to the cell behavior. Thus, by evaluating the cell responses on each respective axis, the heatmaps directly provide insights into under which conditions the secondary or tertiary parameters, in this specific example topography and stiffness, impact cell behavior.

**Figure 4:**
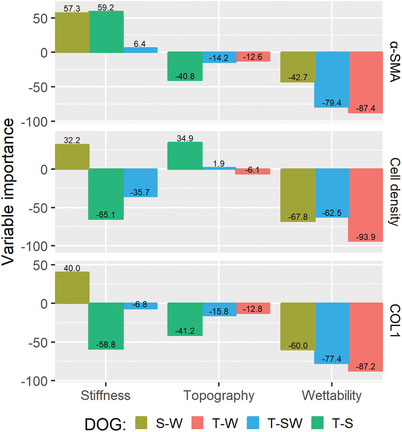
Partial least squares analysis. The relative variable importance (in %, shown on the y‐axis) of the surface stiffness (MPa), topography (in µm), and wettability (water contact angle, WCA, in) (shown on the x‐axis) was plotted for the assessed cell behavior (cell density in cells mm^−2^, α‐SMA CTCF, and COL1 CTCF) for each of the DOGs. Positive values indicate a positive correlation, while negative values indicate a negative correlation. The total of each DOG is 100%.

A partial least squares (PLS) analysis was performed per DOG to find relationships between the surface parameters (topography, stiffness, and wettability) and the cell response behavior (cell density, α‐SMA, COL1). This analysis allows to assess the relative importance of the combinations of surface parameters within each DOG separately instead of the dataset as a whole (**Figure** **4**). In this way, it can be observed whether the observed trends are dependent on the other parameters they are combined with. The contributions of each surface parameter (variable) relative to the other variables were assessed, where the total influence of the parameters would always be 100 (Figure 4 and y axis). Data on the R‐squared values and number of components are shown in Figure S8 (Supporting Information), providing the % of the variance that can be explained by the model. Similar trends can be observed as found from the RF model feature importance, as it is clearly visible that in each of the DOGs containing a wettability gradient (S‐W, T‐W, and T‐S|W), wettability remains the strongest factor in determining cell density, α‐SMA, and COL1 expression. This correlation is also clearly visible on scatterplots of all the combined data (Figure S7, Supporting Information). The importance of stiffness appears to depend on the other parameters it is combined with, as the variable importance of stiffness on α‐SMA on the S‐W DOG is high (57.3%), whereas it is low (6.4%) on the T‐S|W DOG. Moreover, on the DOG with a fixed hydrophilic surface (T‐S) it appears that both topography and stiffness play a role, although it is worth noting that the R‐squared value of the PLS on this DOG is low, especially in the case of α‐SMA (Figure S8a, Supporting Information). Overall, the PLS analysis shows that the relative importance of each parameter varies based on which other parameter it is combined with, and it also varies between the three different biological outputs of cell density, α‐SMA and COL1 CTCF.

Combined, material properties can affect cell adhesion and fibrosis markers α‐SMA and COL1, depending on their specific combination of parameters. Trends were observed within specific DOGs that show that wettability is the most influential factor, while the impact of stiffness and topography can vary based on their combinations with other surface properties, which can therefore be used to finetune the cell behavior.

### 2.4 Translation of Physicochemical Properties Toward Larger Substrates Substantiate Screening Findings

**Figure 5:**
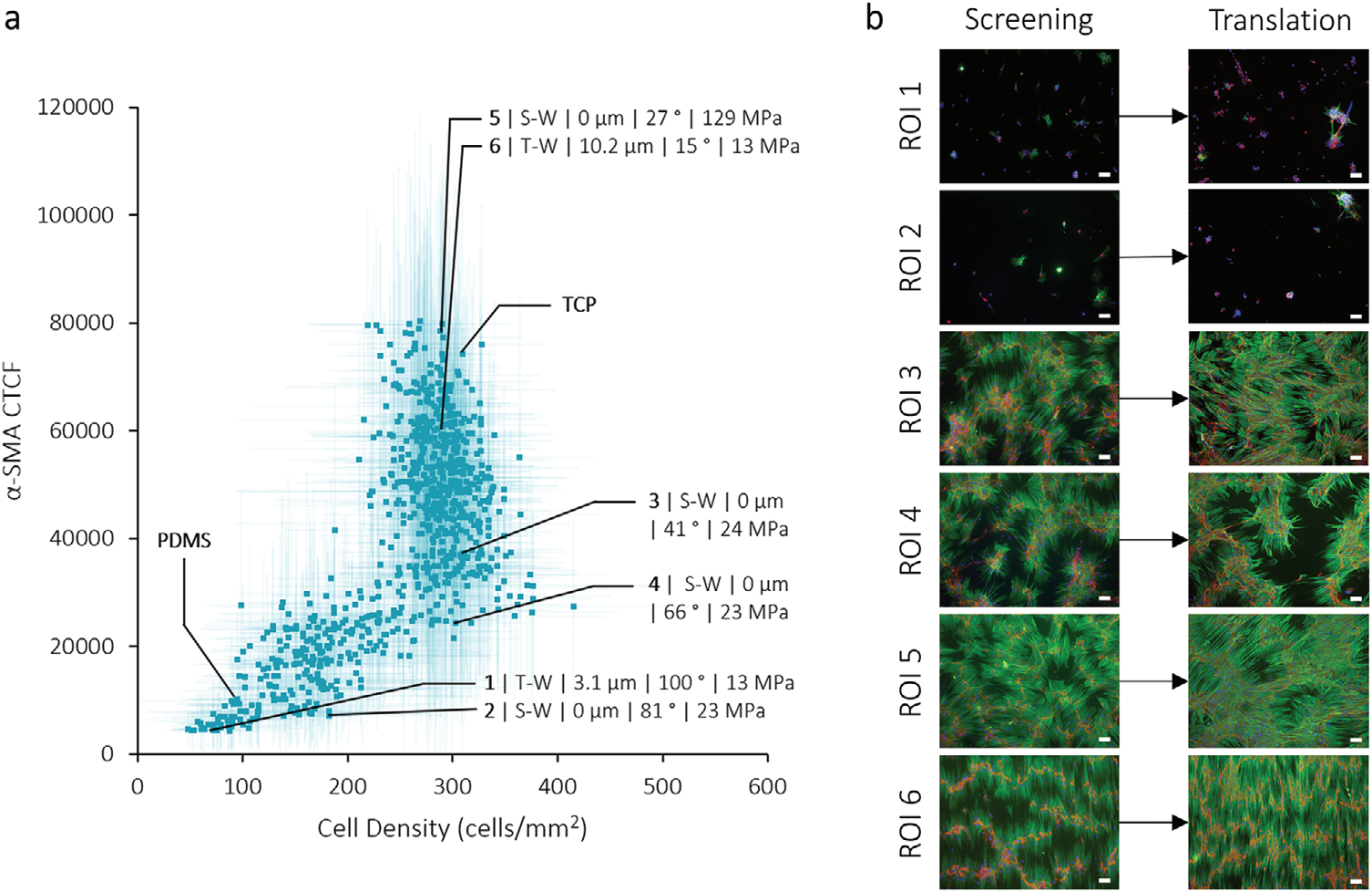
ROI selection and translation to homogeneous surfaces. a) Scatter plot showing all datapoints of the DOGs cell density (cells mm^−2^) versus α‐SMA (CTCF). ROIs 1 to 6 are indicated, which were further used for translation experiments. b) Fluorescence images of the ROIs from the screening and the comparison with the translation experiments. Cells were stained for nucleus (blue), α‐SMA (green), and COL1 (red). Scale bar is 100 µm.

Regions of interest (ROIs) were chosen according to three classes (Figure 3 and **Figure** **5**a): low cell density and low α‐SMA expression (ROI 1 and 2), high cell density and intermediate α‐SMA expression (ROI 3 and 4), high cell density and high α‐SMA expression (ROI 5 and 6). These ROIs were used to investigate points that could be interesting for translation toward implants and verify the screening results. Although α‐SMA expression was chosen as the marker to decide which datapoints to incorporate, it is worth noting that the trends correspond for COL1 expression as well.

From the in vitro screening, ROIs 1 and 2 could be interpreted as the most promising for the prevention of fibrosis due to their reduction in α‐SMA expression, but they also exhibit a very low cell density. When observing the trends between cell density and α‐SMA expression, two distinct patterns can be identified (Figure 5a). The lower region of the graph indicates that as cell density increases, so does α‐SMA expression. However, as cell density reaches a plateau ≈300 cells mm^−2^, α‐SMA continues to increase. This suggests a maximum of the adhered fibroblasts to the surface, while the transformation into myofibroblasts varies. In order to decrease fibrosis development, our objective was to identify material property combinations that are able to support cell adhesion for possible integration of an implant into the surrounding tissue while maintaining a lower level of α‐SMA expression (ROIs 3 and 4), albeit still elevated compared to untreated PDMS.

**Figure 6:**
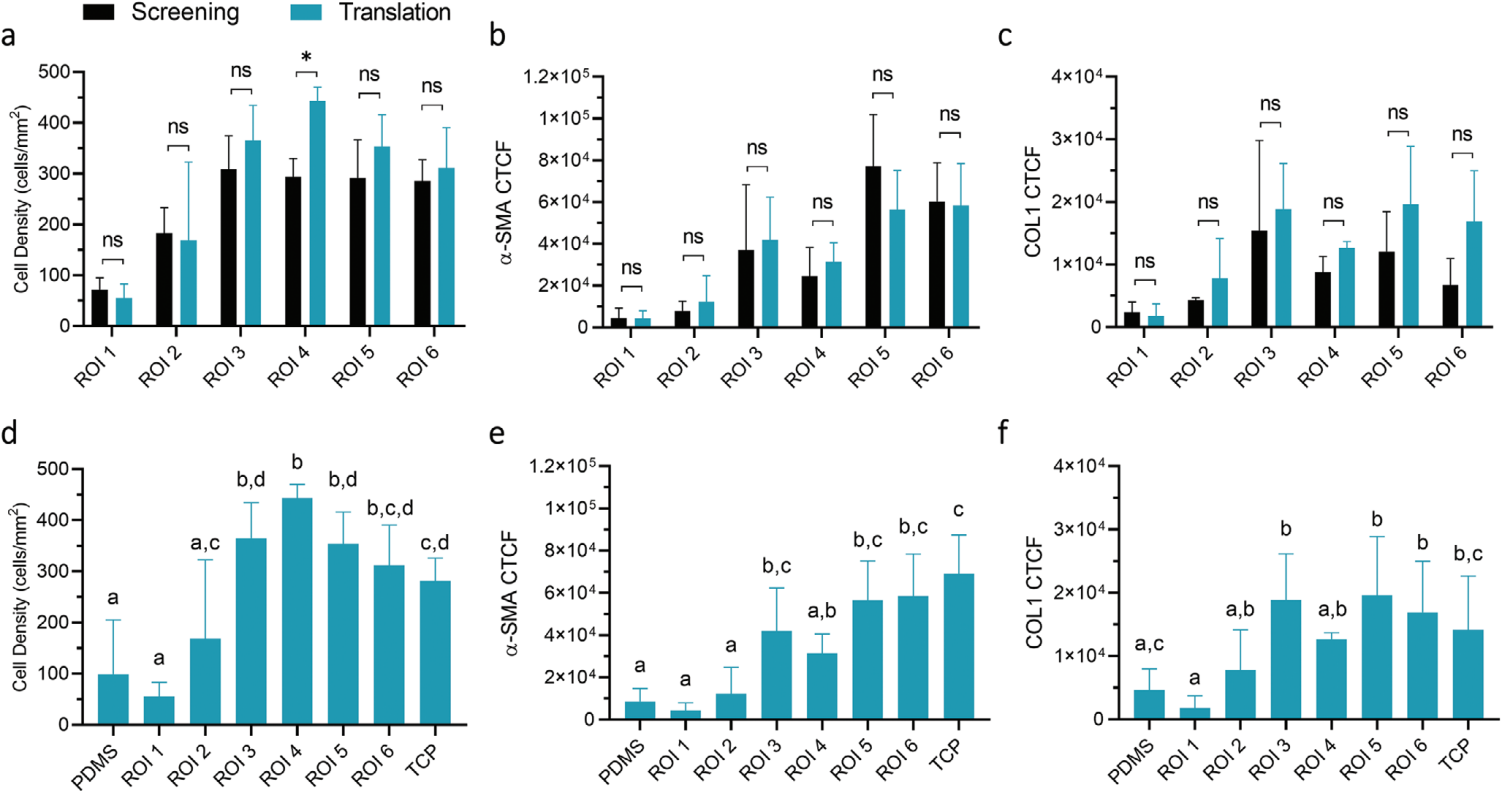
Quantification of comparison screening and translation experiments after 7 days TGF‐β (+). a–c) Screening and translation cell density (cells mm^−2^), α‐SMA (CTCF), and COL1 (CTCF) were compared for statistically significant differences (p<0.05). d–f) Results of ROI translation (n = 3) were compared and tested for statistical significance. All bars sharing the same letter do not have a significant difference between the groups (p>0.05). Groups not sharing a letter indicate a statistically significant difference (p<0.05).

It is important to verify the screening results originating from the gradient samples, as they could be affected by cell migration or cell‐cell communication with different locations on the gradient. The same cell study was therefore repeated on homogeneous substrates to eliminate those effects. In order to translate the exact combination of material properties present on the ROIs to a uniform sample, treatment parameters were carefully identified and used to generate homogeneous substrates bearing those desired properties (Figure S9, Supporting Information). These were used to repeat the cell experiments and the results were compared to the original screening findings (Figure 5b and **Figure** **6**).

The original quantified values from the screening were compared to those of the translation experiments (Figure 6 for 7d TGF‐β (+), Figure S10, Supporting Information for 7d TGF‐β (‐)). As shown in Figure 6a–c, the comparative analysis shows that the cell behavior in terms of cell density, α‐SMA, and COL1 expression was consistent for almost all regions, suggesting a reliable validation of the screening data. ROIs 1 and 2 show a cell density of 100–150 cells mm^−2^, combined with low α‐SMA and COL1 expression, with no statistically significant difference between the screening outcome and translated homogeneous substrate. ROIs 3–6 all indicate high cell density of > 250 cells mm^−2^, aligning well between the screening and translation data. However, a slight disparity is noted in ROI 4, where the translation data shows an even higher cell density compared to the screening.

By comparing the results between ROIs (Figure 6d–f), it can be noted that ROIs 1–2 correspond to a low cell density / α‐SMA / COL1 expression, similar to untreated PDMS, while ROIs 5–6 correspond to a high cell density / α‐SMA / COL1 expression, similar to the results found on TCP. ROIs 3–4 display an increased cell density compared to the “low regions” untreated PDMS and ROIs 1–2. Although, ROI 3 shows an increased α‐SMA and COL1 expression compared to these “low regions”, ROI 4 does not show any significant difference with the “low regions”, while differing significantly from TCP, suggesting a true intermediate phenotype between those “low” and “high” regions.

Summarized, ROIs were selected based on cell density and α‐SMA expression, representing low, intermediate, and high responses. The results of the screening were verified by repeating them on homogeneous substrates bearing those specific combinations of material properties, which shows consistent cell behavior across different ROIs, with ROIs 3 and 4 identified as promising for reducing fibrosis.

## 3 Discussion and Conclusion

This study focused on the early‐stage transformation of fibroblasts toward myofibroblasts, a crucial process during fibrosis development around medical implants. We investigated the influence of combined physicochemical material properties on the early myofibroblast transformation using the BiomACS technology.^[^ ^60^ ^]^ Cells cultured on the platform in the absence of TGF‐β resulted in minimal myofibroblast transformation irrespective of the surface parameters, indicating that the range of material properties present in the DOGs alone are not sufficient to trigger a fibrotic response within the initial 7 days of cell culture. Although most studies focus on TGF‐β‐dependent fibroblast‐myofibroblast transition,^[^ ^16^, ^62^ ^]^ a study by Vogel et al. found transformation toward myofibroblasts without the addition of TGF‐β due to the tensile forces generated by their cell‐generated tissue environment in engineered clefts.^[^ ^63^ ^]^ The mechanisms underlying cellular response to tensile forces may differ from those influenced by changes in stiffness, wettability, and topography, which do not seem to affect fibroblast transformation in the absence of TGF‐β. In contrast to the findings without TGF‐β, varying surface material properties in the presence of TGF‐β were able to generate a wide range of myofibroblast transformations over a 7‐day period, as indicated by their α‐SMA and COL1 expression. A random forest machine learning model and PLS analysis were able to identify the relative importance of each parameter, of which wettability played the most significant role. Screening ROIs were validated by repeating the experiments on homogeneous substrates, confirming consistent trends in cell behavior. Our results of “high adhesion” ROIs 5–6 align well with previous findings, stating that highly adhesive silicone surfaces trigger severe fibrotic capsule formation and contracture, which is mainly driven by the activation of myofibroblasts.^[^ ^34^ ^]^ However, the contribution of macrophages is equally important to the development of fibrosis.^[^ ^14^, ^64^ ^]^ Our findings with untreated hydrophobic PDMS and ROIs 1–2 indicate low fibroblast adhesion and low α‐SMA expression after 7 days, which would appear favorable for preventing fibrosis. However, over longer time periods, chronic inflammation induced by immune cells, particularly macrophages, could still induce fibrotic capsule formation, as reported previously.^[^ ^13^, ^34^ ^]^ Since the non‐adhesive PDMS leaves a “dead space” between the implant surface and the surrounding tissue, non‐specific absorption of blood plasma and serum proteins can trigger the activation of macrophages. In turn, these activated macrophages secrete pro‐fibrotic cytokines, initiating the activation of myofibroblasts and ultimately resulting in capsule formation.^[^ ^14^, ^44^ ^]^ Recognizing this phenomenon, we aimed to identify regions that do facilitate cell adhesion while minimizing the transition of fibroblasts toward myofibroblasts. As part of the normal wound healing response, the adhered cells would mask the implant surface for the immune system, thereby preventing the material to be recognized as foreign.^[^ ^65^, ^66^ ^]^ This consequently could stop the inflammatory response, and lead to proper integration into the surrounding tissue. Our strategy was therefore to identify ROIs with an increased cell density while remaining in the lower range of α‐SMA expression, which could prevent the aggravated development of fibrosis. ROI 4 offers such an intermediate phenotype and should therefore be investigated further. Development of fibrosis during the FBR is a complex, multi‐stage process that is a complicated interaction between multiple cell types, including fibroblasts and macrophages.^[^ ^67^ ^]^ While myofibroblasts differentiation is a critical step toward developing fibrosis, it does not fully represent the complexity of fibrosis as it excludes other key players and its interactions. Our results cultured without the supplementation of TGF‐β demonstrate that fibroblasts need more cues in their environment than only the presence of the material properties, as no significant myofibroblast differentiation is observed. Hence, the inflammatory environment containing cytokines secreted by for example immune cells is needed to orchestrate the full fibrotic response, which cannot be completely mimicked in vitro. The findings of this screening must undergo verification to assess the long‐term impact on macrophages and other players during the FBR before being able to translate toward medical implants. For future studies, in vivo evaluation of fibrosis will provide a more relevant and complex environment to assess the impact of the specific material properties of the ROIs found in this screening on the overall fibrotic response. These studies should include the role of multiple cell types, such as macrophages, and more relevant clinical outcomes such as capsular contracture. However, the results from the current study already indicate that our screening method is an effective tool for detecting changes in fibroblast behavior, providing valuable insights into how biomaterial properties can influence the differentiation process ultimately leading to fibrotic responses.

The importance of each parameter on cell behavior was quantified using different methods, by performing both a machine‐learning random forest regression model and a PLS analysis. While the PLS analysis assumes a linear relationship between the predictor and response variables, the random forest regression was able to also define non‐linear relationships, leading to higher model accuracy from our dataset. However, despite the different strengths and weaknesses in each approach, our findings regarding feature importance between the two methods align well. In general, wettability was found to have the greatest influence on cell behavior, followed by stiffness.

These findings regarding the impact of each parameter on cell behavior can be elucidated through comparison with existing literature on the subject. From this study, wettability arose as the predominant factor in determining myofibroblast behavior. The mechanism by which wettability impacts cell adhesion involves the alteration of the protein adsorption on the surface.^[^ ^60^, ^68^ ^]^ Proteins that are crucial for cell adhesion, such as fibronectin and vitronectin, may undergo conformational changes depending on the surface's wettability. In turn, this affects the ligands that are present for integrins, cell surface receptors that mediate cell adhesion and signaling.^[^ ^69^ ^]^ The conformation of the proteins bound to the surface can either enhance or inhibit integrin binding and the subsequent signaling pathways that could lead to myofibroblast differentiation. An optimal surface wettability enables enough protein adsorption with conformations that are accessible for cell attachment.^[^ ^68^ ^]^ Focal adhesion formation through integrins can promote the actin cytoskeleton development, generating contractile forces and potential myofibroblast differentiation.^[^ ^70^ ^]^ Previous studies have shown that a moderately hydrophilic surface of ≈70° is most optimal for in vitro culture of fibroblasts.^[^ ^71^ ^]^ More recently, Park et al. studied the effect of oxygen (O_2_) plasma modification of smooth, micro‐ and nanotextured implants in rats, and reported decreased capsule formation on the treated implants.^[^ ^72^ ^]^ They also note a decrease in TGF‐β expression, which could indicate that the more hydrophilic surface leads to a different immune response, which in turn leads to less activated fibroblasts. Our results indicate high myofibroblast activation on hydrophilic surfaces in the presence of TGF‐β, but without the stimulation of TGF‐β, no fibrotic response is observed. The next step of this research would therefore be to include players from the immune system, such as macrophages, to investigate the effect of the identified material properties on the inflammation.

On the influence of stiffness on cell behavior, the ECM is known to alter physical processes to influence stem cell fate.^[^ ^73^ ^]^ Cells are able to sense and respond to changes in their environment by cellular processes such as mechanosensing,^[^ ^74^, ^75^ ^]^ chemotaxis,^[^ ^76^ ^]^ and topotaxis.^[^ ^77^, ^78^ ^]^ Within fibroblast‐mediated matrix remodeling, cells were shown to express more α‐SMA on stiff plates compared to collagen lattices. Moreover, the researchers conclude the implication of α‐SMA in contraction and remodeling of the ECM.^[^ ^79^ ^]^ Stiffness can mechanically activate pro‐fibrotic TGF‐β, resulting in fibrotic encapsulation. This was reported by a study of Hinz et al., indicating that by adding a softer silicone layer on top of the stiff silicone, less collagen deposition and myofibroblast activation was found.^[^ ^31^ ^]^ However, our study uses an initially stiff silicone substrate, and we investigated the effect of surfaces even stiffer. Even within these elevated stiffness ranges, we were able to identify a moderate effect of stiffness on α‐SMA expression.

Based on our results, change of the wrinkle size of the surface topography was not directly correlated to the cell density, α‐SMA, and COL1 expression. Previous studies have also investigated the impact of topography on the FBR.^[^ ^1^, ^32^, ^38^, ^80^ ^]^ For example, Langer et al. studied how the surface roughness of silicone breast implants influences the FBR.^[^ ^1^ ^]^ Their research demonstrated that of different implants with a surface roughness of 0–90 µm, a surface roughness of ≈4 µm induced minimal inflammation and FBR in different animal models and humans. Our screening, however, did not find such as strong influence of topography on the in vitro transformation of myofibroblasts. This discrepancy may be attributed to the overshadowing effect of the additional parameters such as wettability and stiffness. It is worth noting that changes in surface topography can also impact the wettability of the surface. However, topography‐mediated wettability is a macroscopic phenomenon and in a completely wetted system, surface polarity is the main driver in wettability while topography then connects to cell behavior purely via a mechano‐sensing effect. When studying the effects of multiple parameters on cell responses, it is crucial to consider potential confounding factors that may influence the observed outcome.^[^ ^81^ ^]^ Addition of topography to a hydrophobic surface (WCA > 90⁰) can increase the apparent hydrophobicity by trapping air pockets underneath the topography on which the water droplet will rest. If a surface is hydrophilic (WCA < 90⁰), the apparent hydrophilicity can increase as the water droplet adapts to the presented surface topography.^[^ ^82^ ^]^ However, in all these situations it is the gas phase, usually air, that induces the enhanced effect and it is absent in a completely wetted system as cell culture where no air is present anymore at the solid‐liquid interface. The effect of topography‐related wettability changes are therefore of little consequence for cell behavior but rather the mechano‐sensing effects of cells toward that topography. Besides, the utilization of DOGs enables to alter each parameter separately on each axis of the sample. Therefore, even if there are topography‐related wettability changes on the axis of the topography gradient, the effect of the wettability gradient by the plasma oxidation treatment can be evaluated independently by only comparing points of similar topography, along the direction of the wettability gradient axis. Our results additionally show a contribution of topography with specific combinations of the other properties of stiffness and wettability, as datapoints in favorable regions are not solely on flat PDMS but also on the wrinkled substrates. Therefore, once we focus on specific ranges of favorable wettability and stiffness, the addition of topography could finetune the cell response to a favorable outcome, albeit not based on a specific trend but more on defined “hits”. Besides that, the complexity of the FBR can also introduce other factors not accounted for in our in vitro experiments. Additionally, roughness may not directly be comparable to the anisotropic wrinkled topography as used in this screening. More general studies have looked into fibroblast adhesion and spreading to surfaces with different topography, indicating that addition of fibers or microgrooves, initiate cell alignment and reduce cell spreading.^[^ ^83^, ^84^, ^85^ ^]^ On smooth surfaces, fibroblast tend to spread out randomly, while on textured surfaces (such as grooves or fibers), cells align along the direction of the topographical cues.^[^ ^86^ ^]^ This alignment was also observed visually in our results, although it did not result in different myofibroblast transformation as measured by α‐SMA and COL1 expression. Therefore, it would be of great interest to investigate which processes during the first hours of cell adhesion and spreading ultimately lead to those differences in myofibroblast transformation as observed in our screening and ROI translation experiments.

Although the current focus lies on silicone‐based materials, extending this approach to materials with different chemical composition or modifications is feasible, depending on the application of interest.^[^ ^87^ ^]^ For instance, the topography gradient used in the current screening approach has already successfully been adapted for use with various inorganic materials,^[^ ^88^ ^]^ and there is a potential for further exploration with a broader range of materials. Moreover, including surface functionalization by chemical modification would be of great interest and could expand the screening method even further beyond mechanical properties. For example, Wang et al. recently introduced a novel high‐throughput screening method based on rational design and statistical learning to functionalize surfaces with peptides,^[^ ^89^ ^]^ demonstrating that materials can be modified in many ways that have yet to be fully explored. Therefore, the general technique of the BiomACS screening platform utilized in this research can provide insights in cell‐material interactions wider than just silicone breast implants, fibroblasts, or the foreign body response. Optimal biomaterial surfaces can be easily screened with in vitro experiments, depending on the biological question, leading to a more rapid advancement and possibilities to move to clinical studies. Within implant design optimization, it can aid in the discovery of material properties that steer the behavior of host cells, immune cells, and even microorganisms and thereby have an impact on future medical device performance in vivo.

In conclusion, we performed a high‐throughput screening of varying topography, stiffness, and wettability on the differentiation of fibroblasts into fibrotic myofibroblasts. We were able to reveal wettability as predominant factor influencing cell behavior, followed by stiffness. Translational experiments on homogeneous substrates bearing specific material property combinations of interest were able to validate the screening findings, highlighting regions with increased cell adhesion and reduced myofibroblast differentiation, offering the potential for implant surface optimization to prevent fibrosis.

## 4 Experimental Section

### DOG Fabrication

**Figure 7:**
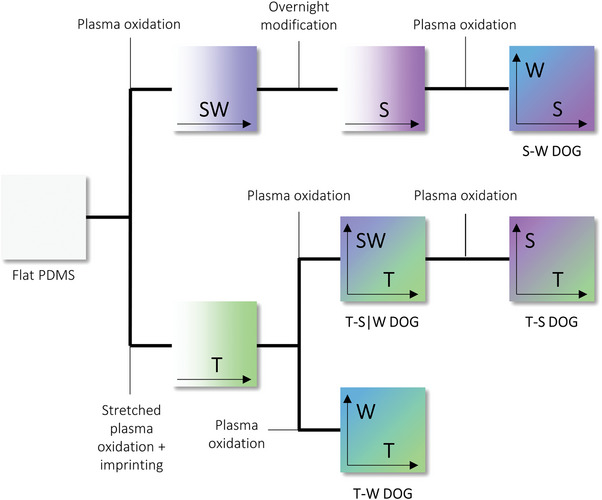
Schematic overview of sequential plasma treatments of polydimethylsiloxane (PDMS) substrates, resulting in four different double orthogonal gradient (DOG) surfaces to be used for cell screening experiments.

The DOG screening platform was configured into four distinct samples, each containing two surface parameter gradients positioned orthogonally while maintaining a fixed third parameter. Every DOG was created by combining topography (T), stiffness (S), and wettability (W) gradients through shielded plasma oxidation treatments applied to PDMS substrates, as shown in **Figure** **7** and described in previous methods.^[^ ^58^, ^59^, ^60^ ^]^ Each treatment was elaborated upon in the following sections.

### PDMS Preparation

PDMS films were prepared using a commercially available two‐component kit (DowDuPont). In brief, elastomer (Sylgard‐184A) and curing agent (Sylgard‐184B) were mixed to a ratio of 10:1. The mixture was poured into a 12×12 cm square petri dish. After spreading and degassing, the PDMS was cured at 70 °C overnight.

### Topography (T) Gradient

The wrinkled topography (T) gradient was prepared using previously published methods.^[^ ^86^, ^90^, ^91^ ^]^ PDMS films were cut into 3.5×3.5 cm samples and placed into a custom‐made stretching device. The sample was stretched by 20% of its original length and covered with a right‐angled triangular prism mask 2.2 cm long and 2 cm wide with an angle of 30°. Then, the sample was oxidized with air plasma (Diener Electronic, model Atto, Ebhausen, Germany) at 25 mTorr for 650 s at maximum power. A wrinkled topography gradient was formed on the surface upon slow release of the stress on the PDMS sample. These PDMS wrinkle gradients were fully oxidized with a second air plasma treatment of 150 mTorr for 600 s and used as a mold. A fresh mixture of elastomer/curing agent (10:1) was poured on the molds, followed by curing at 70 °C overnight. The molds were removed, and the remaining topography (T) imprints were used for further experiments.

### Double Linear Stiffness‐Wettability (S|W) Gradient

To create a double linear stiffness‐wettability (S|W) gradient, PDMS films were partially covered with a right‐angled triangular prism mask of 2 × 2 cm with a 30° angle and treated with air plasma oxidation at 40 mTorr for 20 s at maximum power.^[^ ^92^ ^]^ The plasma treatment makes the surface stiffer and more hydrophilic, increasing both parameters from the closed to the open side. To generate the double orthogonal gradient of topography‐stiffness/wettability (T‐S|W), this treatment was performed on top of the topography (T) gradient imprints, orthogonally to the first gradient. To generate the double orthogonal gradient of topography‐stiffness (T‐S), this T‐S|W sample was fully oxidized at a treatment at 1000 mTorr for 20 s to render the surface fully hydrophilic, with only the topography and stiffness gradient remaining.

### Stiffness (S) Linear Gradient

The stiffness (S) gradient was isolated from the stiffness‐wettability (S|W) gradient by placing the samples into a desiccator containing a vial with 50 µL trichloro(propyl)silane (99%, TCI). A vacuum was applied overnight to yield a hydrophobic monolayer on the PDMS surface. The samples were rinsed with 70% ethanol to remove the remaining contaminants.

### Double Orthogonal Stiffness‐Wettability (S|W) Gradient

A second plasma treatment was performed orthogonal to the single stiffness (S) linear gradient to create the double orthogonal stiffness‐wettability (S|W) gradient. This was done using the previously described 2 × 2 cm 30° mask using a treatment at 1000 mTorr for 20 s at maximum power. This milder treatment affects only the hydrophilicity of the surface without affecting the stiffness.

### Wettability (W) Linear Gradient

In order to create the double orthogonal gradient of topography‐wettability (T‐W), a mild plasma treatment was performed on top of the topography gradient imprints, orthogonally to the first gradient, using a treatment at 1000 mTorr for 60 s. This milder treatment affects only the hydrophilicity of the surface without affecting the stiffness.

### Topography Characterization

The wrinkled surface topography was measured using a NanoScope V Dimension 3100 atomic force microscope (Bruker, Billerica, MA, USA) with corresponding NanoScope Analysis software. Tip DNP‐10 with cantilever “D” (Bruker) was used in air contact mode to scan the surface's topographical features. Measurements were performed at 11 points in the direction of the topography gradient, with the 11th point located outside of the prism mask, yielding the largest wrinkle size. Three measurements were performed per position perpendicular to the direction of the gradient. The wavelength and amplitude of the wrinkles were determined in NanoScope Analysis software using the Section tool.

### Stiffness Characterization

The surface stiffness was measured using a Catalyst NanoScope V instrument equipped with an RTESPA‐150 tip (tip radius = 8 nm, tip half angle = 18°). Tip calibration was performed on a reference glass slide to determine the tip spring constant (k = 2 to 3 N/m). Peakforce Quantitative Nanomechanical mapping mode was used to determine the DMT modulus over a preset surface area of 500 nm^2^. 5 points were measured along the stiffness gradient, with three individual measurements per position.

### Wettability Characterization

The surface Water Contact Angle (WCA) was characterized by an in‐house developed tensiometer. Using the sessile drop method, droplets of 5 µL Milli‐Q water were placed on the surface. The WCA was determined by a MATLAB (MathWorks) program that measures the interfacial WCA between the surface and the droplet. Five measurements were taken along the gradient direction, with three measurements per position. The WCA measurements were performed directly after plasma oxidation.

### Cell Culture

Normal Human Dermal Fibroblasts (NHDFs, C‐12302, PromoCell, passage 4) were maintained in High Glucose Dulbecco's Modified Eagle Medium (HG DMEM, Gibco) containing 10% (v/v) fetal bovine serum (FBS, Gibco) and 1% penicillin/streptomycin (p/s/, Gibco). Cell cultures were maintained at 37 °C and 5% CO_2_.

### Cell Culture on Double Orthogonal Gradients

Wettability gradients were prepared directly before proceeding to cell culture and stored in Milli‐Q. The DOG samples were cut into circular pieces of ⌀34 mm to fit into the well of a 6‐well plate. Samples were sterilized with 70% ethanol for 10 min and washed twice with sterile phosphate‐buffered saline (PBS) for 5 min. The samples were stored in PBS until cell seeding. Cells were harvested with 0.05% trypsin‐EDTA (Gibco), seeded at a density of 5000 cells cm^−2^ directly on the DOG samples, and incubated at 37 °C and 5% CO_2_. After 8 h, the medium was changed to serum starvation medium (HG DMEM) containing 0.5% FBS and 1% p/s, with or without 0.17 mmol L^−1^ vitamin C (ascorbic acid, AA) and 10 ng mL^−1^ TGF‐β1 (Peprotech).

### Immunofluorescence

For immunofluorescence of α‐smooth muscle actin (αSMA) and collagen type I (COL1), the cells were fixed and stained after 24 h and 7 days. In brief, the cells were washed with PBS and fixated with ice‐cold methanol/acetone (1:1) for 20 min at −20 °C. The fixed cells were washed with PBS and permeabilized with 0.5% Triton X‐100 (Sigma–Aldrich/Merck) in PBS. Non‐specific binding sites were blocked with 10% bovine serum albumin (BSA) for 30 min. Next, cells were incubated with a primary antibody solution of mouse anti‐aSMA (Abcam, ab7817, 1:500) and rabbit anti‐collagen type I (Abcam, ab138492, 1:1000) in 2.2% BSA in PBS for 1 h. Cells were washed three times with 0.05% PBS‐Tween (Sigma–Aldrich), and incubated with a secondary antibody solution of goat anti‐mouse FITC (Jackson Immuno Research, 1:100), donkey anti‐rabbit Alexa Fluor 647 (Jackson Immuno Research, 1:500), and DAPI (2 µg mL^−1^) in 2.2% BSA in PBS for 1 h. Cells were washed twice with 0.05% PBS‐Tween, once with PBS, and stored in PBS in the dark at 4 °C until imaging.

### Visualization and Analysis

Imaging was performed using the automatic scanning Zeiss AxioObserver.Z1 TissueFAXS microscope (TissueGnostics, Vienna, Austria). The complete DOG area (20×20 mm^2^) was imaged at 10X magnification and images were combined with TissueGnostics software. Image analysis was performed using the open‐source software CellProfiler. A pipeline was developed to perform automated cell segmentation, including removal of large artefacts, and analysis of the different image channels. From each image (1742×1298 µm) located on the 20×20 mm^2^ area, the median cell density, and α‐SMA and Collagen type I corrected total cell fluorescence (CTCF) were calculated. CTCF was defined as the integrated density minus the cell area (in µm^2^) times the mean background intensity.

To investigate a correlation between α‐SMA expression and surface topography, stiffness, and wettability, a machine‐learning model was trained with 10‐fold cross‐validation using a random forest regression algorithm. The Random Forest Regression module was described in Jupyter Notebook version 6.5.4 using Python programming language. 75% of the data was used for model training and validation, while 25% of the data was held out to test the model. Model training was performed 100 times with random splitting of the data, and the average R^2^ value was determined.

### Region of Interest (ROI) Translation

ROIs 1–6 were translated toward homogeneous substrates using the following treatments: 1) untreated imprint of λ = 3 µm; 2) 1000 mTorr 7 s on flat PDMS; 3) 1000 mTorr 15 s on flat PDMS; 4) 1000 mTorr 11 s on flat PDMS; 5) 40 mTorr 6 s on flat PDMS; 6) 1000 mTorr 18 s on imprint of λ = 10 µm. Imprints of λ = 3 and 10 µm were prepared by 25 mTorr 60 s at 30% stretch and 25 mTorr 10 min at 20% stretch, respectively, followed by an imprint in fresh 10:1 PDMS. Cell culture studies were repeated for these ROIs, an untreated PDMS control, and a TCP control, as described before.

### Statistical Analysis

Partial least squares regression was performed on the data of 7 days with stimulation of TGF‐β the individual DOGs using R version 4.3.1 and the “pls” package version 2.8.2. PLS regression was performed on each DOG using three dependent variables (topography, stiffness, and wettability) and three independent variables (cell density, α‐SMA and COL1). Each variable was scaled by dividing by its standard deviation. In the cases of the T‐S, T‐W, and S‐W DOG, which have one fixed parameter, an offset term was added to avoid numerical errors due to a standard deviation of zero. Leave‐one‐out (LOO) cross‐validation was used to determine the number of components to consider. Plots were created with the R package “ggplot2” version 3.4.2.

Data represented was expressed as the mean values from the replicates (n = 4 for 7 days TGF‐β (+), n = 2 for 7 days TGF‐β (‐) and for 24 h, n = 3 for translation experiments). Statistical analysis was performed using GraphPad Prism 8.0.1. For comparison of the 50 lowest and 50 highest values from the screening, a two‐way analysis of variance (ANOVA) was performed, to determine differences between the groups. To compare the findings from the screening and the translation, a two‐way ANOVA was performed with Sidak's multiple comparisons test. To compare the ROIs from the translation experiment, an ordinary one‐way ANOVA with Tukey's multiple comparisons test was performed. A p‐value of < 0.05 was considered significant.

## Conflict of Interest

P.v.R. is also co‐founder, scientific advisor, and share‐holder of BiomACS BV, a biomedical oriented screening company.

## Supporting information



Supporting Information

## Data Availability

The data that support the findings of this study are available from the corresponding author upon reasonable request.
