# Supplementary material for: Modulation of Biomaterial‐Associated Fibrosis by Means of Combined Physicochemical Material Properties
Source: Adv Sci (Weinh). 2024 Dec 6;12(4):2407531. doi: 10.1002/advs.202407531 (PMC11789587; doi:10.1002/advs.202407531)
Supplement: Supplementary file 1 — Supporting Information [file ADVS-12-2407531-s001.pdf]

ADVANCED SCIENCE
Open Access

Supporting Information

for *Adv. Sci.*, DOI 10.1002/advs.202407531

Modulation of Biomaterial-Associated Fibrosis by Means of Combined Physicochemical Material Properties

*Lisa E. Tromp, Torben A.B. van der Boon, Roderick H.J. de Hilster, Ruud Bank and Patrick van Rijn**

Supplementary Information

Modulation of Biomaterial-Associated Fibrosis by Means of Combined Physicochemical Material Properties

Authors:

Lisa E. Tromp, Torben A.B. van der Boon, Roderick H.J. de Hilster, Ruud Bank, Patrick van Rijn

Affiliations

Lisa E. Tromp, University of Groningen, University Medical Center Groningen, department of Biomaterials and Biomedical Technology FB-40, A. Deusinglaan 1, 9713 AV, Groningen, the Netherlands. Email: l.e.tromp@umcg.nl. ORCID ID: 0000-0003-0346-0072

Torben A.B. van der Boon, University of Groningen, University Medical Center Groningen, department of Biomaterials and Biomedical Technology FB-40, A. Deusinglaan 1, 9713 AV, Groningen, the Netherlands. Email: t.a.b.van.der.boon@umcg.nl ORCID ID: 0000-0003-3705-2800

Roderick H.J. de Hilster, University of Groningen, University Medical Center Groningen, department of Biomaterials and Biomedical Technology FB-40, A. Deusinglaan 1, 9713 AV, Groningen, the Netherlands. Email: r.h.j.de.hilster@umcg.nl ORCID ID: 0000-0002-6931-2323

Ruud Bank, University of Groningen, University Medical Center Groningen, department of Pathology and Medical Biology, A. Deusinglaan 1, 9713 AV, Groningen, the Netherlands. Email: r.a.bank@umcg.nl

Patrick van Rijn, University of Groningen, University Medical Center Groningen, department of Biomaterials and Biomedical Technology FB-40, A. Deusinglaan 1, 9713 AV, Groningen, the Netherlands. Email: p.van.rijn@umcg.nl ORCID ID: 0000-0002-2208-5725

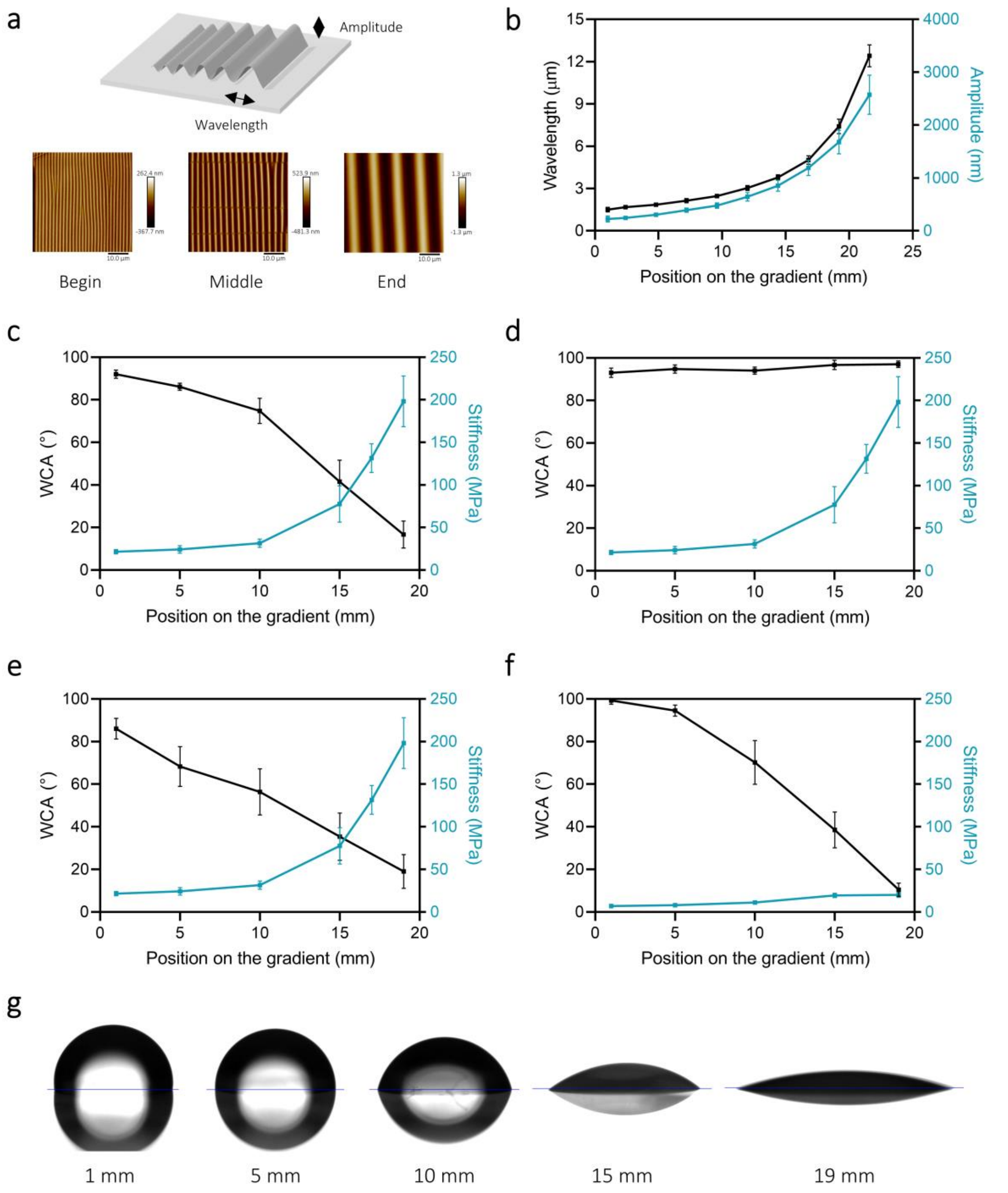

Figure S1 Gradient characterization. a, Surface topography gradient characterization with increasing wavelength and amplitude from the closed to the open side of the triangular prism mask. **b,** Quantification of the wrinkle wavelength and amplitude by AFM. **c**, The coupled plasma treatment affects both the wettability (Water Contact Angle, WCA) and stiffness (MPa) resulting in a S|W linear gradient. **d**, Silanization of the S|W linear gradient of **c** leads to a hydrophobic monolayer resulting in an isolated S gradient. **e**, Addition of a second uncoupled treatment to **d** affects only the wettability, resulting in the S-W DOG. **f**, The uncoupled plasma treatment affects only the wettability and leads to a single W gradient. **g**, WCA images of the coupled plasma treatment shown in **c**.

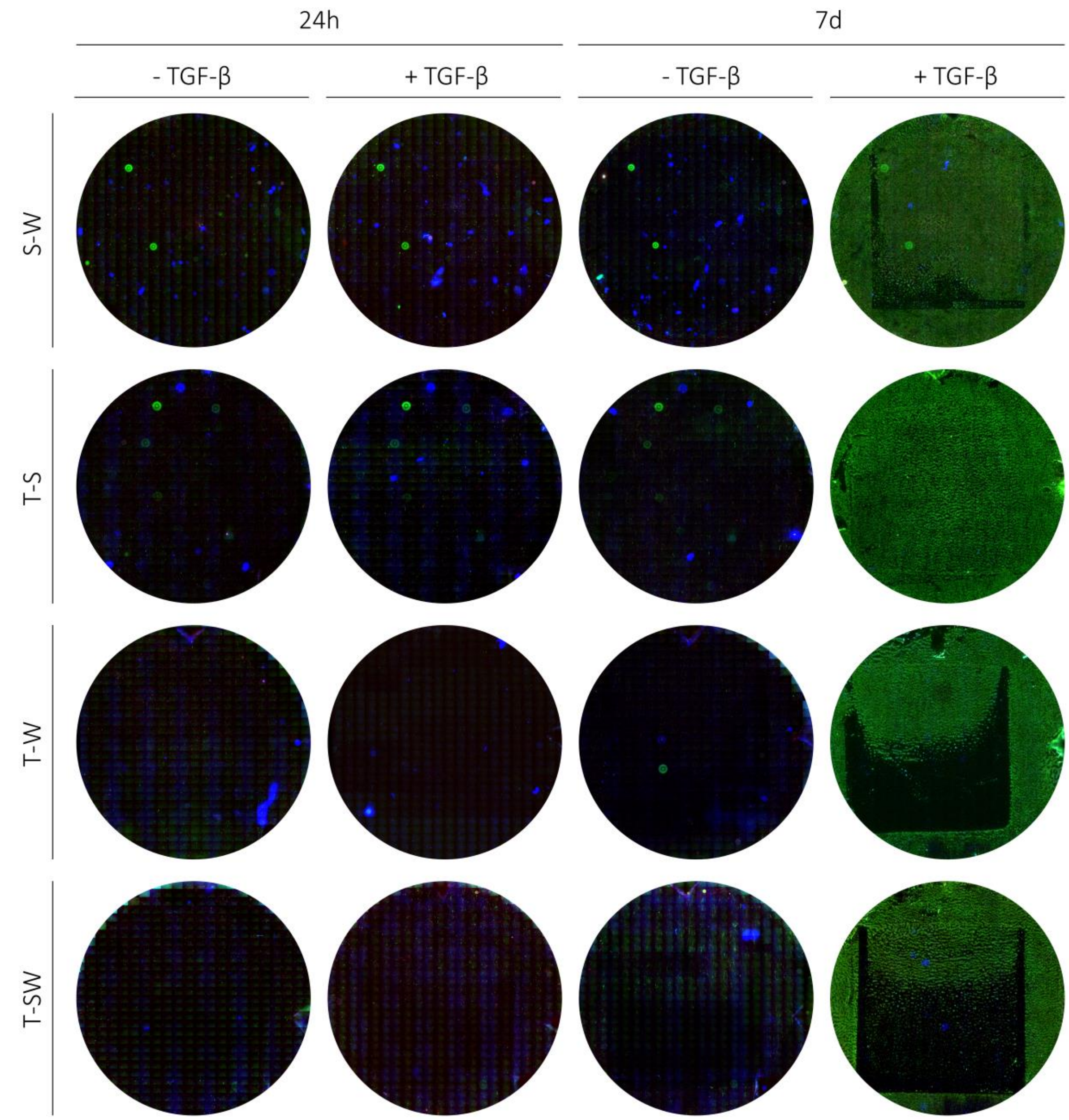

Figure S2 Overview of the stitched images from the TissueFAXs automated fluorescence microscope. Each DOG region is 20 x 20 mm in size. DAPI is shown in blue, Collagen type I in red, and α-SMA in green.

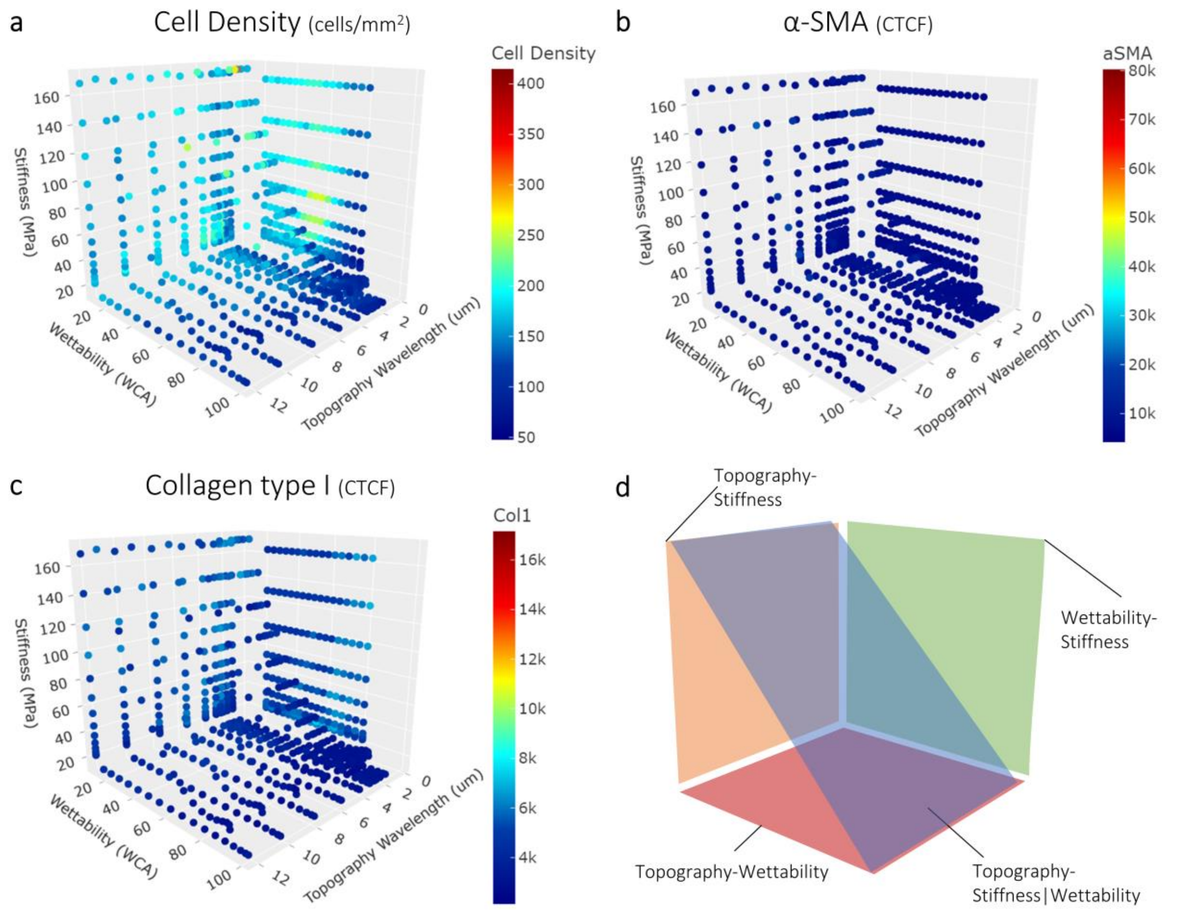

Figure S3 DOG screening overview after 7 days without stimulation of TGF-β. Three-dimensional plots show the mean of the screening replicates (n=2) as a color scale on the axes of topography, stiffness, and wettability. **a**, Cell density is quantified as cells/mm2. **b**, α-SMA expression was quantified as Corrected Total Cell Fluorescence (CTCF, a.u.). **c**, Collagen type I expression was quantified as CTCF (a.u.). **d**, Schematic overview of each plane in the three-dimensional box, corresponding to each DOG.

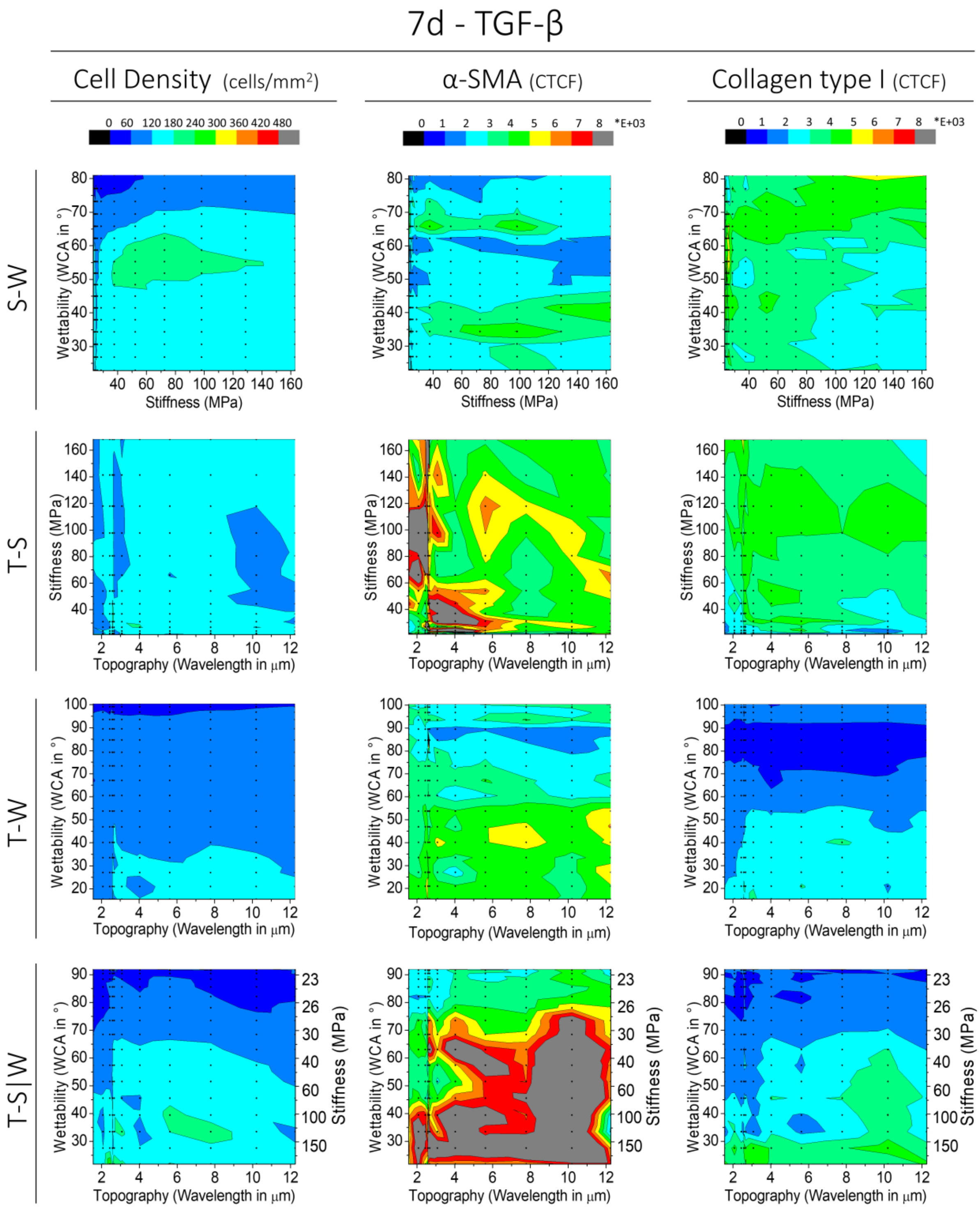

Figure S4 Heatmaps showing the cell screening results after 7 days without stimulation of TGF-β on each DOG. The average value (n=2) of cell density in cells mm^{-2}, α-SMA CTCF, and COL1 CTCF was shown on each of the DOGs.

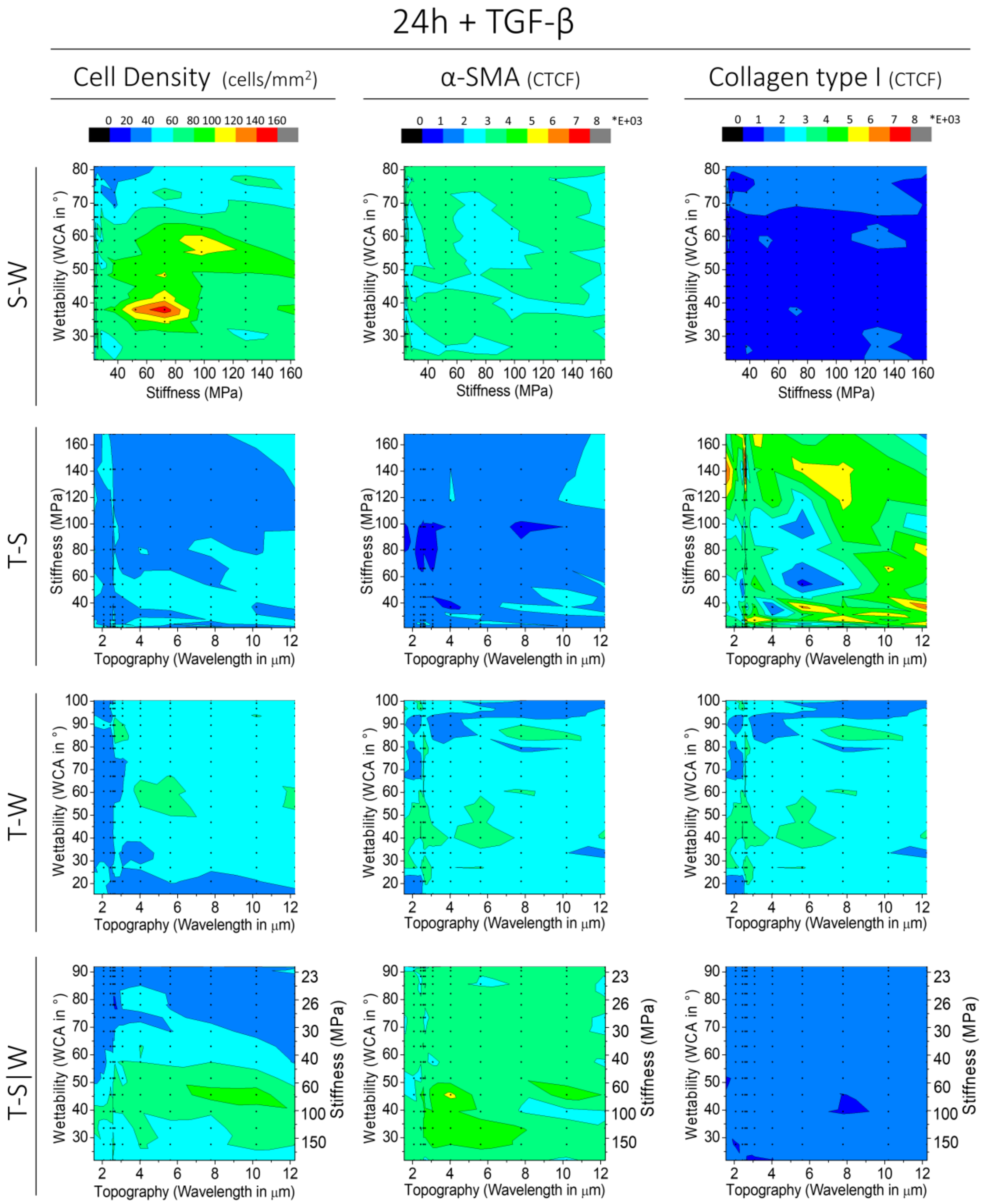

Figure S5 Heatmaps showing the cell screening results after 24 hours with stimulation of TGF-β on each DOG. The average value (n=2) of cell density in cells mm^{-2}, α-SMA CTCF, and COL1 CTCF was shown on each of the DOGs.

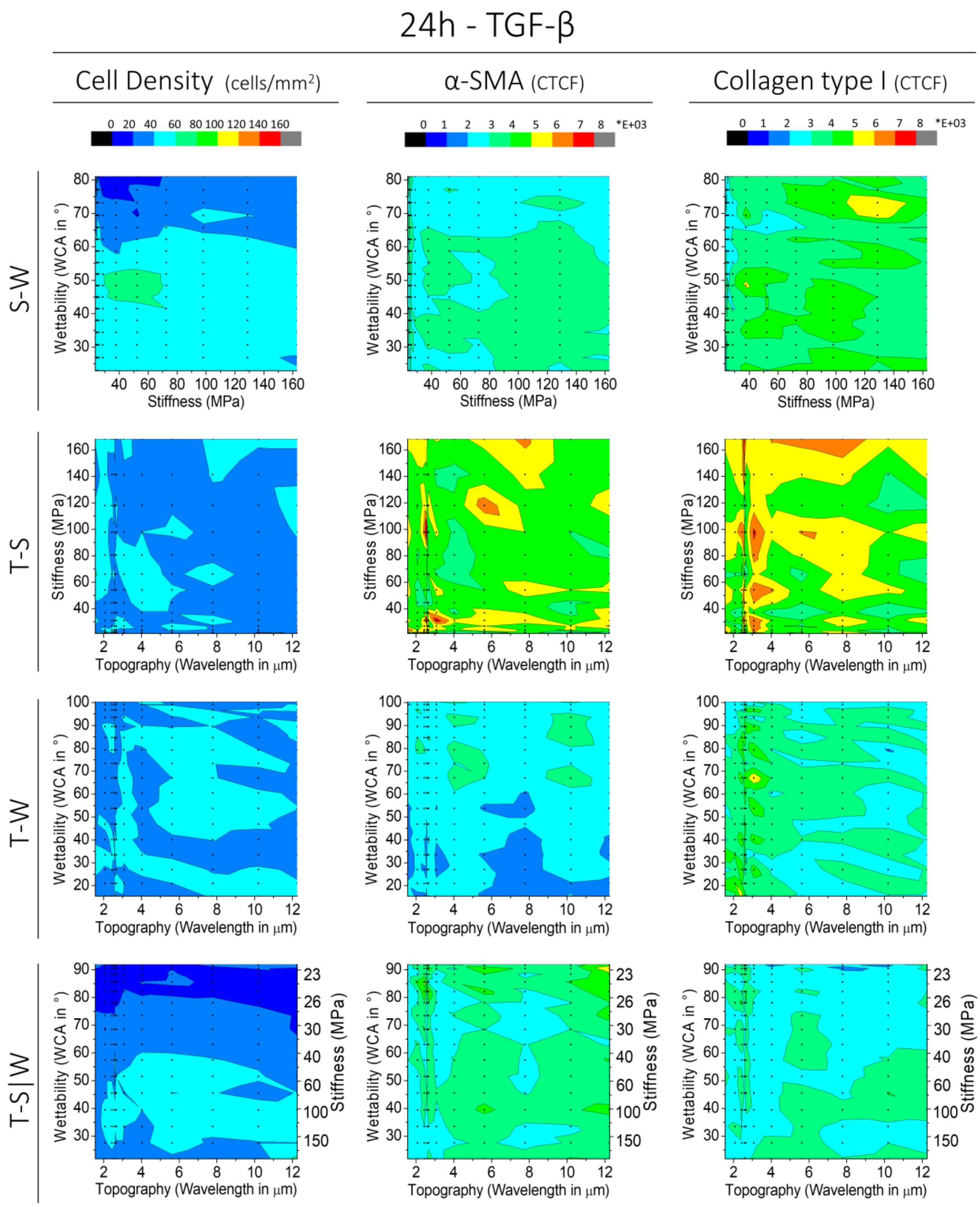

Figure S6 Heatmaps showing the cell screening results after 24 hours without stimulation of TGF-β on each DOG. The average value (n=2) of cell density in cells mm^{-2}, α-SMA CTCF, and COL1 CTCF was shown on each of the DOGs.

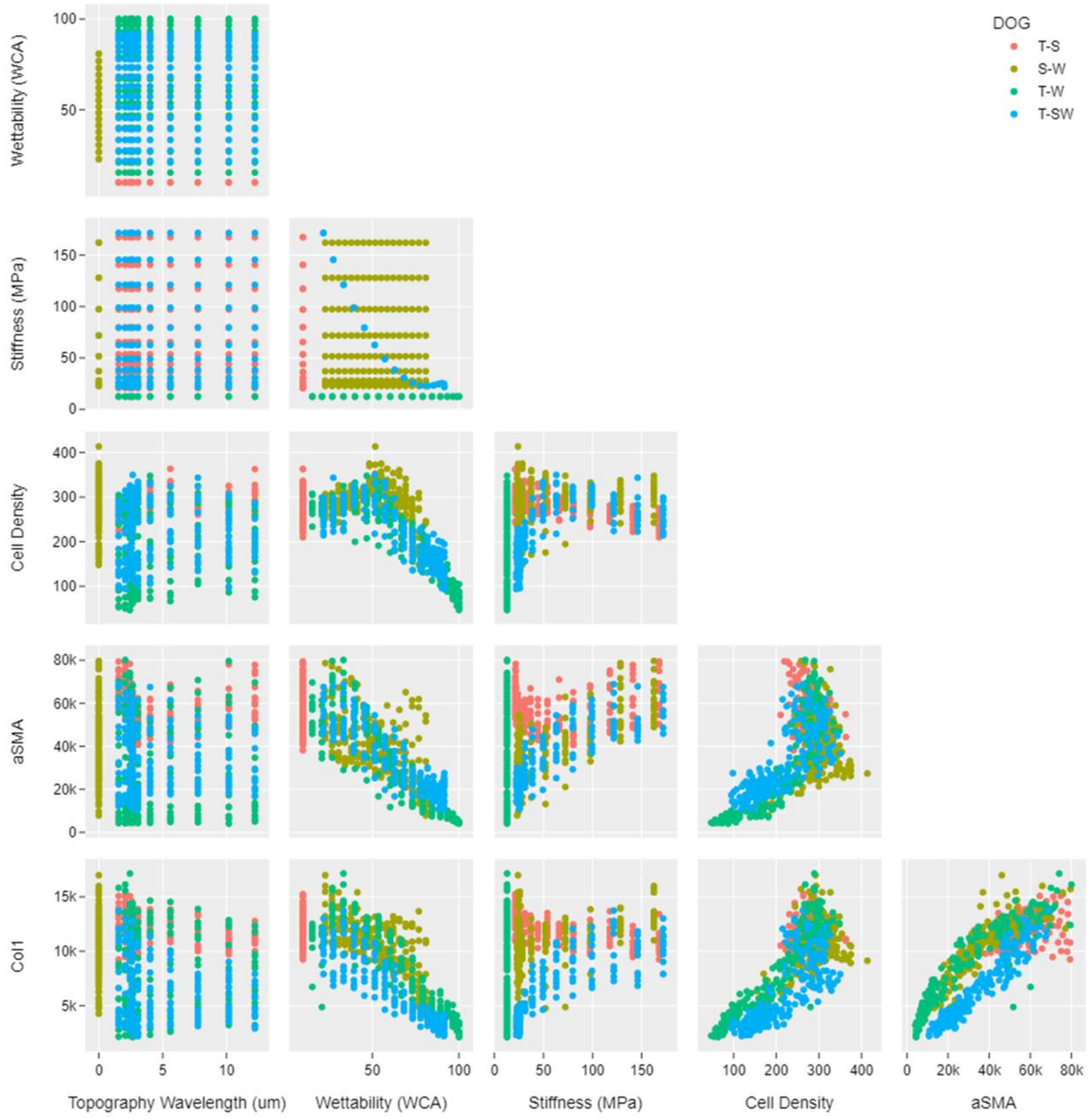

Figure S7 Overview of the complete dataset (7 days with TGF-β) in scatterplots that indicate the relationships between topography, stiffness, wettability, cell, density, α-SMA CTCF, and COL1 CTCF. The different DOGs are indicated with different colors.

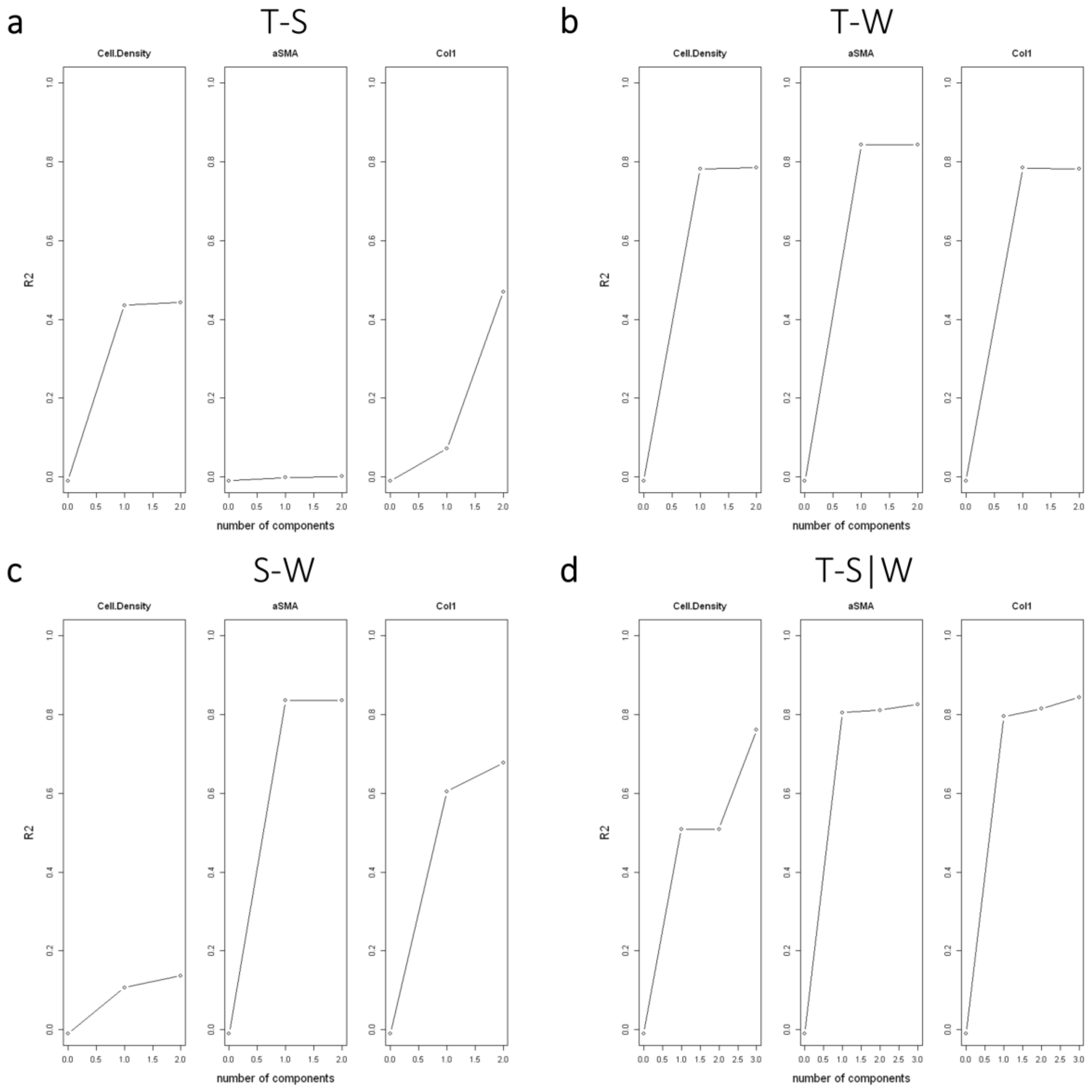

Figure S8 R-squared values and number of components used for the partial least squares regression. In each case, the highest number of components was used to describe the data, as they provide the best R^2 value.

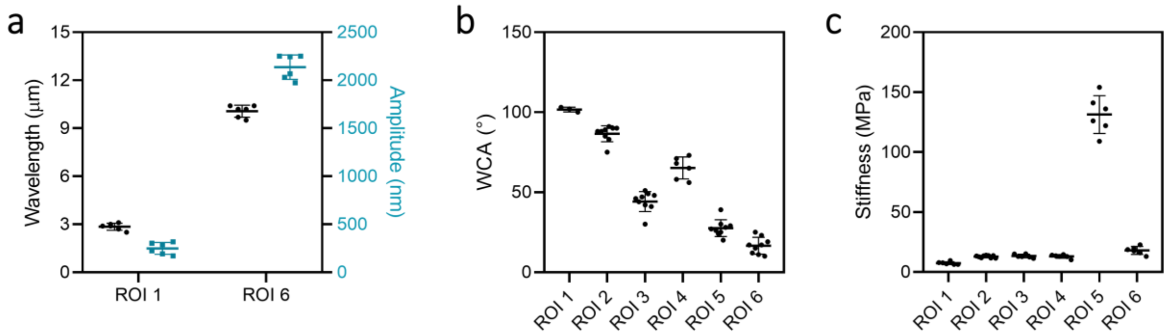

Figure S9 Characterization of ROIs.a, Measurement of the topography wavelength in µm and amplitude in nm for ROI 1 and 6, other ROIs were flat. **b**, Water contact angle (in °) measurements of ROIs. **c**, Stiffness (Young's modulus in MPa) measurements of ROIs.

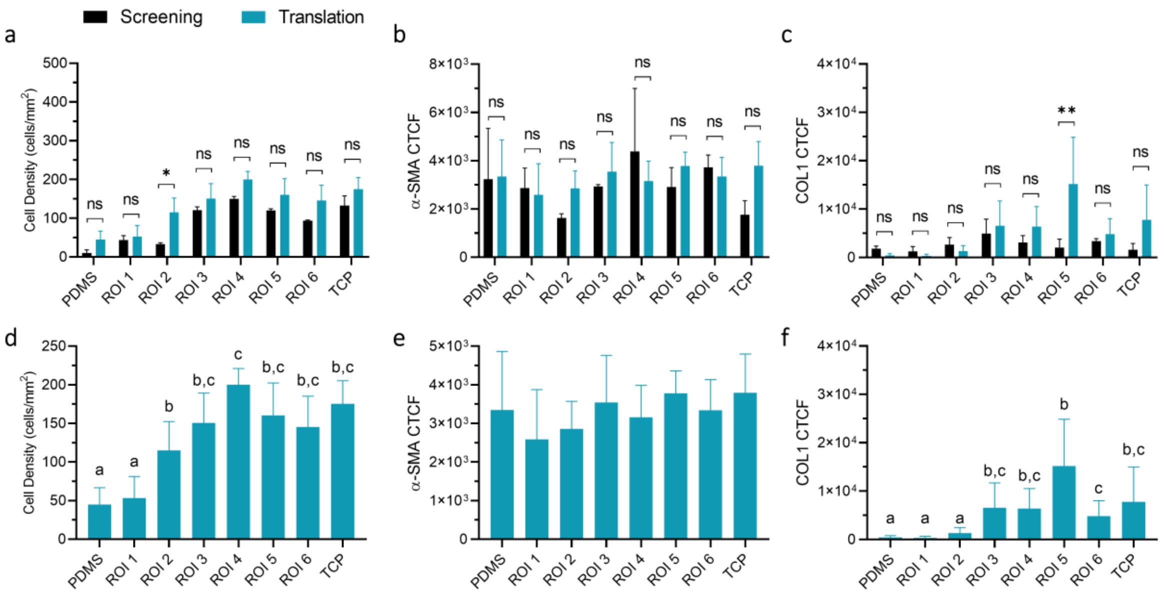

Figure S10 Quantification of comparison screening and translation experiments after 7 days TGF-β (-). a-c, Screening and translation cell density (cells mm^{-2}), α-SMA (CTCF), and COL1 (CTCF) were compared for statistically significant differences ($p<0.05$). **d-f**, Results of ROI translation (n=3) were compared and tested for statistical significance. All bars sharing the same letter do not have a significant difference between the groups ($p>0.05$). Groups not sharing a letter indicate a statistically significant difference ($p<0.05$).
